# Clinical efficacy and safety of adjunctive treatment of chronic ischemic heart failure with Qishen Yiqi dropping pills: a systematic review and meta-analysis

**DOI:** 10.3389/fcvm.2023.1271608

**Published:** 2023-12-18

**Authors:** Wang Xingmeng, Dai Guohua, Guan Hui, Gao Wulin, Qu Huiwen, Fan Maoxia, Li Runmin, Ren Lili

**Affiliations:** ^1^ The First School of Clinical Medicine, Graduate School of Shandong University of Traditional Chinese Medicine, Jinan, Shandong, China; ^2^ Department of Geriatrics, Affiliated Hospital of Shandong University of Traditional Chinese Medicine, Jinan, China; ^3^ College of Traditional Chinese Medicine, Shandong University of Traditional Chinese Medicine, Jinan, China

**Keywords:** Qishen Yiqi dripping pills, chronic ischemic heart failure, prognosis, clinical efficacy, readmission, meta-analysis

## Abstract

**Objectives:**

Our study was to evaluate the effect of Qishen Yiqi Dropping Pills(QSYQ) on the prognosis of chronic ischemic heart failure(CIHF) and its safety.

**Methods:**

Databases including CNKI, Wanfang, VIP, CBM, PubMed, Web of Science, The Cochrane Library and EMbase were searched from their inception to April 2023 to screen relevant randomized controlled trials (RCTs). Primary indicators included readmission rates, rates of major adverse cardiovascular events (MACE), and all-cause mortality rates. The quality of the literature was assessed according to the Cochrane Reviewers' Handbook 5.0 and the Modified Jadad Scale (with a score of 4–7 rated as high quality). Meta-analysis was performed using the meta-package created by R software version 4.2.3, continuous data were compared using SMDs, and dichotomous and ordered data were compared using ORs; and the *I*^2^ test was used to assess the heterogeneity.

**Results:**

Fifty-nine studies out of 1,745 publications were finally included, totalling 6,248 patients. Most studies were poorly designed and had some publication bias, with only 26 high-quality papers (Jadad score ≥4). Meta-analysis showed that the combined application of QSYQ was able to reduce the readmission rate [OR = 0.42, 95% CI (0.33, 0.53), *P* < 0.001], all-cause mortality rate [OR = 0.43, 95% CI (0.27, 0.68), *P* < 0.001], and the incidence of MACE [OR = 0.42, 95% CI (0.31, 0.56), *P* < 0.001]. Also, the treatment method can improve clinical effectiveness [OR = 2.25, 95% CI (1.97, 2.58), *P* < 0.001], increase 6-min walking distance (6MWD) [SMD = 1.87, 95% CI (1.33, 2.41), *P* < 0.0001] and left ventricular ejection fraction (LVEF) [SMD = 1.08, 95% CI (0.83, 1.33), *P* < 0.0001], and decrease the Minnesota Living with Heart Failure Questionnaire (MLHFQ) scores [SMD = −2.03, 95% CI (−3.0, −1.07), *P* < 0.0001], BNP levels [SMD = −2.07, 95% CI (−2.81, −1.33), *P* < 0.0001] and NT-ProBNP levels [SMD = −2.77, 95% CI (−4.90, −0.63), *P* < 0.05]. A total of 21 studies (*n* = 2,742) evaluated their adverse effects, of which 13 studies reported no adverse effects and 8 studies reported minor adverse effects.

**Conclusion:**

Our results suggest that the combined application of QSYQ can further improve patients' cardiac function and exercise tolerance, improve their quality of life, and ultimately improve patients' prognosis with a favorable safety profile. Nonetheless, limited by the quality and high heterogeneity of the literature, we must be conservative and cautious about the present results.

**Systematic Review Registration:**

PROSPERO (CRD42023449251).

## 1. Introduction

Heart failure (HF) is a serious manifestation and advanced stage of various cardiovascular diseases and is a serious life-threatening complex clinical syndrome (1). Epidemiologic data show that the global prevalence of HF in adults is 1%–3%, but the prevalence is expected to increase in the future due to the aging of the population and the use of effective evidence-based therapies to prolong the lives of patients with HF (2). Even with long-term treatment with internationally standardized medications, patients still suffer from recurrent exacerbation of symptoms such as dyspnea, edema, and fatigue as well as poor quality of life, and mortality and readmission rates remain high (3). Studies have shown that in a given population, the 1-year risk of death in patients with HF ranges from 15% to 30%, with a 5-year risk of death as high as 75% (2). HF has become a serious public health concern worldwide due to its high morbidity and mortality (4). Among many factors, ischemic heart disease (IHD) represents the etiology of HF in 40% of the global HF population. Several studies have shown that the presence of coronary artery disease is associated with a higher risk of death and a worse prognosis in patients with HF after hospital discharge (2, 5). IHD-induced HF is independently associated with mortality compared with nonischemic causes (6, 7). Therefore, there remains an urgent need to find an adjunctive treatment that can improve quality of life and further effectively reduce re-hospitalization and mortality rates.

Chronic heart failure belongs to the category of “heart failure” in Chinese medicine, and the basic pathogenesis is qi deficiency and blood stasis (8). Benefiting qi, activating blood circulation, and inducing diuresis constitute the mainstays of treatment for it. In recent years, Chinese medicine has received increasing attention in the treatment of HF with its unique theories and remarkable efficacy. QiShen YiQi Drop Pills (QSYQ) is one of the representative Chinese medicinal preparations, which is made of Astragalus mongholicus, Salvia miltiorrhiza, Panax notoginseng, and Dalbergia wood oil, and it is considered to have the effects of benefiting Qi, invigorating blood circulation, and dredging blood vessels. Approved by the State Food and Drug Administration (SFDA) in 2003 (National Drug Approval Number: Z20030139), QSYQ was recommended by the “Guidelines for the Diagnosis and Treatment of Chronic Heart Failure in Traditional Chinese Medicine” for the comprehensive treatment of HF or IHD in the type of qi deficiency and blood stasis (9). Existing systematic evaluations (8, 10, 11) provide evidence-based medical evidence for the clinical application of QSYQ to a certain extent, but there are still many shortcomings: (1) To the best of our knowledge, all systematic evaluations have selected surrogate indicators for efficacy evaluation and lacked clinical endpoint indicators and long-term prognostic indicators, such as readmission rate and mortality. Despite the economic efficiency, sensitivity, and accessibility of alternative indicators in clinical studies which have some clinical value (12), they could not provide the most direct evidence to support the improvement of the long-term prognosis of patients with HF by QSYQ. (2) Newly published high-quality randomized controlled trials were not included. A randomized, double-blind, placebo-controlled trial (CACT-IHF) (13) involving 32 centers in China and including 640 patients with chronic ischemic heart failure (CIHF) has been publicly published, and there is no doubt that this trial will have an unprecedented impact on the clinical evidence-based evaluation of QSYQ. (3) Previous systematic evaluations have not conducted further assessment analyses of CIHF. Given these, we conducted an updated systematic evaluation and meta-analysis that included the CACT-IHF trial and used readmission rate, all-cause mortality, and adverse cardiovascular events as the main evaluation indexes, and explored for the first time the clinical efficacy of the adjunctive treatment of CIHF with QSYQ.

## 2. Materials and methods

This systematic review and meta-analysis followed the Preferred Reporting Items for Systematic Reviews and Meta-Analyses(PRISMA) statement and has been registered in PROSPERO (registration number: CRD42023449251).

### 2.1. Ethics approval and consent to participate

This study did not involve animal or patient experimentation and did not require ethical approval or informed consent from participants.

### 2.2. Inclusion criteria and exclusion criteria

The PICOS principles were strictly followed as the eligibility criteria and the followings are included.

#### 2.2.1. Study type

This study included published randomized controlled trials (RCTs) of QSYQ-assisted treatment of CIHF, both nationally and internationally, which were required to have similar study methods, complete general data and statistical analysis with uniform metrics.

#### 2.2.2. Study object

Diagnostic criteria for chronic heart failure were based on the 2022 AHA/ACC/HFSA Guideline for the Management of Heart Failure (14); previous history of myocardial infarction or revascularization, or diagnosis of coronary artery disease confirmed by coronary angiography; and New York Heart Association (NYHA) functional class II–IV. Patients had balanced comparable baseline data.

#### 2.2.3. Intervention measures

The control group was treated with conventional western medications recommended by international guidelines, including those recommended for HF (14, 15) such as ACEI/ARB, β-blockers, aldosterone receptor antagonists, ARNI, and SGLT-2i, as well as those recommended for coronary artery disease (16), such as aspirin, clopidogrel, ticagrelor, calcium antagonists, nitrates, ivabradine, nicorandil, and trimetazidine. Nonetheless, in the QSYQ group, patients also took QiShen YiQi Drops Pills (manufactured by Tianjin Tasly Pharm. Co., Ltd, taken orally, 0.5 g, TID) apart from the medicines taken by the control group. The conventional western medicines may not be consistently taken for each study, and the only difference between the QSYQ group and the control group was whether or not QSYQ was applied. In addition, neither group took any other medications that might interfere with the assessment indicators.

#### 2.2.4. Exclusion criteria

(1) Repeated reports, studies with inaccurate or incomplete literature; (2) Irrelevant studies such as individual cases or empirical reports; (3) Animal experiments, pharmacological mechanism studies; (4) Guidelines, reviews, and systematic evaluations; (5) Descriptive studies only without clinical controlled trials; (6) Non-randomized controlled trials; (7) Inconsistent study subjects; (8) Inconsistent evaluation indexes.

#### 2.2.5. Outcome indicators

Primary efficacy assessment indicators include ① Re-admission rates (RARs); ② All-cause mortality (ACM); ③ Major adverse cardiovascular events (MACE were defined as cardiogenic death, cardiogenic shock, myocardial infarction, revascularization, and severe arrhythmia, etc.).

Secondary efficacy assessment indicators include ④ The Minnesota Living with Heart Failure Questionnaire (MLHFQ) scores; ⑤ Clinical efficacy rates (CERs): Clinical efficacy assessment criteria were formulated in accordance with the “Guidelines for Clinical Research of New Traditional Chinese Medicines” and the NYHA grading. Clinical efficacy is divided into three categories: Significant: Complete relief of symptoms and signs or improvement of cardiac function by more than 2 levels; Effective: Partial relief of symptoms and signs or improvement of cardiac function by 1 level; Ineffective: No significant improvement or aggravation of signs and symptoms, and improvement of cardiac function by less than 1 level; ⑥ 6-min walking distance (6MWD); ⑦ Left ventricular ejection fraction (LVEF); ⑧ Brain natriuretic peptide(BNP); ⑨ N-terminal prohormone of BNP(NT-pro BNP); ⑩ Left ventricular end-diastolic dimensions (LVEDD); ⑪ Left ventricular end-systolic dimensions (LVESD); ⑫ Left ventricular end-diastolic volume (LVEDV); ⑬ Left ventricular end-systolic volume (LVESV).

⑭ Safety indicators include the incidence of adverse reactions such as itchy skin or rash, nausea, vomiting, and dizziness.

### 2.3. Search strategy

A comprehensive and systematic search from 8 databases was conducted to retrieve RCTs from inception to 04/22/2023. The following databases are included: PubMed, Cochrane Library, Embase, Web Of Science, Wanfang Database, China Scientific Journal Database (VIP), China National Knowledge Infrastructure (CNKI), and China Biology Medicine (CBM). We also attempted to search ongoing RCTs, such as the Chinese Clinical Trial Registry, to ensure a comprehensive and exhaustive collection of literature. Search terms included “QiShen YiQi”, “QiShen YiQi Dripping Pills”, “Heart Failure”, “Cardiac Failure”, “cardiac insufficiency”, “chronic heart failure”, etc., and their synonyms. A search strategy combining medical subject terms and free words was adopted. In addition, we manually searched references of published systematic reviews in order to conduct a comprehensive search for other relevant studies. Also, we provide search strategies about Pubmed (Table 1).

**Table 1:** Search strategy for pubMed.

	Search item
#1	"Heart Failure"[Mesh]
#2	[heart failure(Title/Abstract)] OR [Cardiac Failure(Title/Abstract)] OR [Heart Decompensation(Title/Abstract)] OR [Decompensation, Heart(Title/Abstract)] OR [Heart Failure, Right-Sided(Title/Abstract)] OR [Heart Failure, Right Sided(Title/Abstract)] OR [Right-Sided Heart Failure(Title/Abstract)] OR [Right Sided Heart Failure(Title/Abstract)] OR [Myocardial Failure(Title/Abstract)] OR [Congestive Heart Failure(Title/Abstract)] OR [Heart Failure, Congestive(Title/Abstract)] OR [Heart Failure, Left-Sided(Title/Abstract)] OR [Heart Failure, Left Sided(Title/Abstract)] OR [Left-Sided Heart Failure(Title/Abstract)] OR [Left Sided Heart Failure (Title/Abstract)]
#3	#1 OR #2
#4	[Qishen Yiqi Dripping Pills (Title/Abstract)] OR [Qishen Yiqi Dripping Pill(Title/Abstract)] OR [Qishen Yiqi DropPill(Title/Abstract)] OR [Qishen Yiqi(Title/Abstract)] OR [Qishen Yiqi droplet(Title/Abstract)] OR [Qishen Yiqi Pills(Title/Abstract)]
#5	#3 AND #4

### 2.4. Article selection and data extraction

Based on the inclusion and exclusion criteria, two researchers (Qu HW and Li RM) screened the literature independently and in parallel using EndnoteX9 software to minimize subjective selection bias, and resolved disagreements by consulting a third party member (Wang XM). Studies that clearly did not meet the inclusion criteria were excluded first by reading titles and abstracts. Then the full texts of the remaining studies were carefully read to decide on inclusion or exclusion. Finally, all the included studies were cross-checked to ensure eligibility.

Two researchers (Wang XM and Qu HW) independently and in parallel extracted data including article title, first author, year of publication, country, journal, participant's age, gender, sample size of QSYQ and control groups, intervention, treatment duration, methodological information, efficacy evaluation indexes, and adverse effects. The authors of the original studies were contacted by e-mail or telephone when necessary to obtain the missing but essential information for the studies.

### 2.5. Quality evaluation

The quality of included studies was independently assessed and checked by two researchers (Qu HW and Li RM), and disagreements were resolved through consultation with a third-party person (Wang XM). Assessment was performed using the Cochrane Risk of Bias Assessment Tool (17), which covers seven areas: Randomized sequence generation, allocation concealment, blinding of investigators and subjects, blinding of outcome assessors, completeness of outcome data, selective reporting, and other biases. All of these were assessed as “low risk of bias”, “high risk of bias”, or “unclear risk of bias”. The quality of the studies was evaluated using the modified Jadad scale, which includes four aspects: Randomized sequence generation, allocation concealment, blinding, withdrawal and exit. The scores were 2, 2, 2, and 1, respectively. The quality of RCTs with a score of 1–3 was rated as low, and the quality of RCTs with a score of 4–7 was rated as high.

### 2.6. Statistical analysis

This meta-analysis was performed based on the meta-package (18) created by R software version 4.2.3. The dichotomous data were compared using the odds ratios (OR) values; the continuous data were compared using the standardized mean difference (SMD) due to the differences in participants' cardiac function between studies. To make the best use of the data, a maximum likelihood ratio fitted to the cumulative ratios model was used for the ordinal ranked data and the efficacy categories were described by the odds ratios (OR) and their 95% confidence intervals (95% CI) were calculated. *Z*-tests were used to assess the combined statistical results, and *P* < 0.05 was considered statistically significant. Heterogeneity between studies was assessed using the *I*^2^ statistic and the *χ*^2^-based Cochran *Q* test. When heterogeneity was not significant (*I*^2^ < 50% or *p* > 0.05), a fixed-effects model was used for the combination of effect sizes; otherwise, a random-effects model was used. In addition, we calculated 95% prediction intervals to assess the true range of influence of QSYQ across future studies based on the method recommended by IntHout et al. (19).

The courses of treatment varied among the included studies, and to explore the sources of heterogeneity, we performed subgroup analysis of 6MWD, LVEF, BNP, NT-proBNP, and LVEDD according to the courses of treatment. Multifactorial meta-regression analysis were also performed for 6MWD, and LVEF according to mean age as well as quality of the literature. Then, pooled analysis was further conducted for high-quality (Jadad score ≥4) studies. As heterogeneity remained high across subgroups, the Galbraith plots and Baujat plots were used to identify potential sources of heterogeneity between studies, and the data were recombined after excluding outlier studies. Publication bias was assessed by plotting contour-enhanced funnel plots for indicators that included more than ten studies; Egger's linear regression test was carried out to detect the publication bias in continuous-type data, and Harbord test and Peters' test were implemented to detect the publication bias in dichotomous data (20). If publication bias was detected (*P* < 0.05), contour-enhanced funnel plots were trimmed and filled to explore the causes of funnel plots asymmetry, and effect sizes were recombined for the corrected funnel plots. Sensitivity analysis was also performed to analyze the robustness of the results.

## 3. Results

### 3.1. Characteristics of the included studies

**Figure 1:**
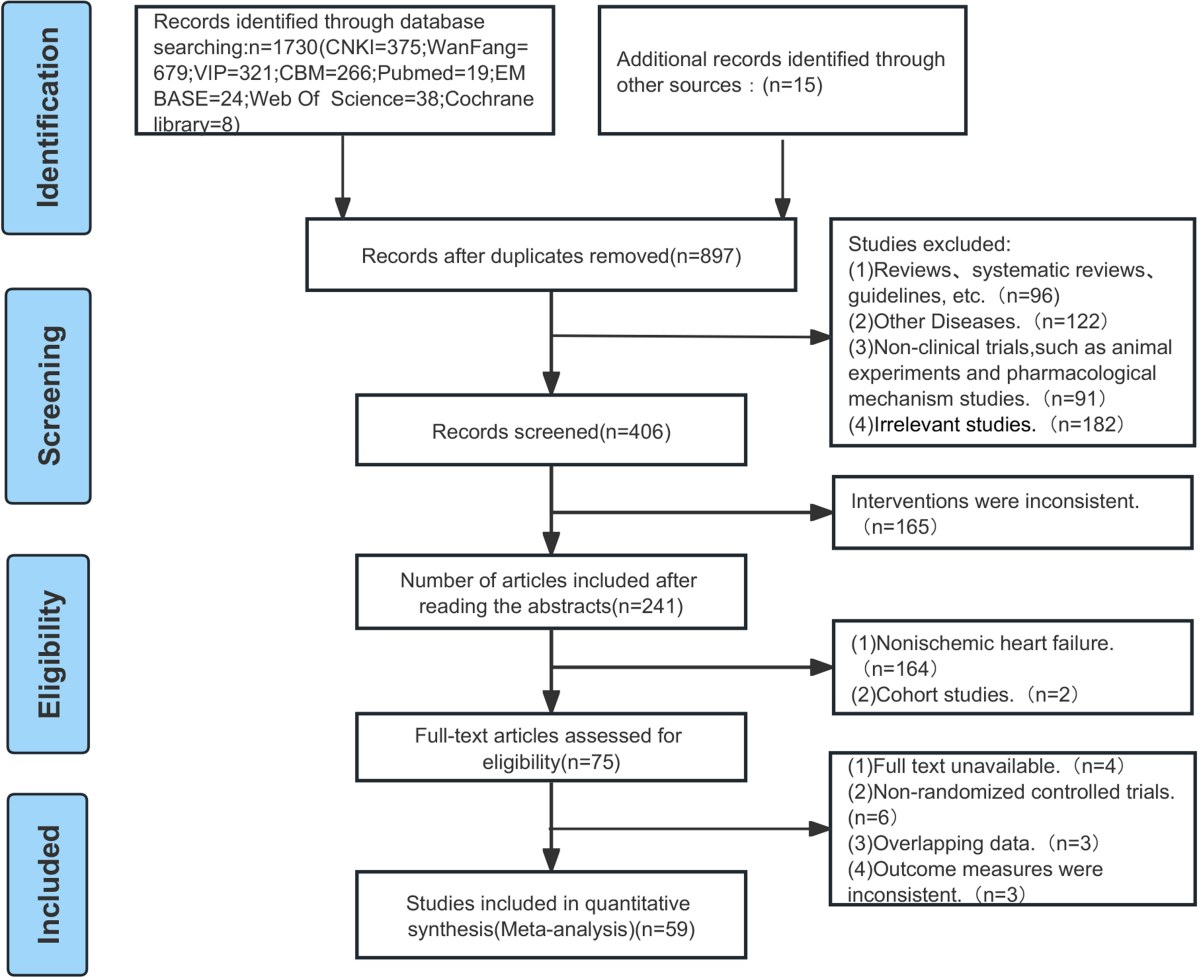
Flow diagram of literature screening.

A total of 1,745 papers were retrieved from 9 databases. After eliminating duplicates (*n* = 848), 656 papers were excluded by reading titles and abstracts. Then, the full text of the remaining 241 papers was read through to exclude the studies with the following inadequacies: non-ischemic heart failure (*n* = 164), cohort studies (*n* = 2), non-randomized controlled trials (*n* = 6), overlapping data (*n* = 3), inconsistent outcome metrics (*n* = 3), and unavailability of full text (*n* = 4). Fifty-nine papers (13, 21–78) were ultimately included for meta-analysis, involving 6,248 patients with CIHF who met the criteria. The literature screening flowchart is shown in Figure 1.

Of the 59 included studies, only one (13) was published in English and provided the largest sample size (*n* = 640), while the remaining 58 were published in Chinese, with sample sizes ranging from 40 to 300; both males and females participated in the studies, with a mean age range of 53.9–86 years. Among the studies involving prognostic indicators, the duration of follow-up ranged from 8 weeks to 48 weeks, with most of the studies focusing on 24 weeks or 48 weeks, but no studies with follow-up longer than 48 weeks. The majority of patients had a cardiac function classification falling within the NYHA class II–III. Supplementary Table S1 (Supplementary materials) summarizes the basic characteristics of the 59 studies.

### 3.2. Quality evaluation of included studies

**Figure 2:**
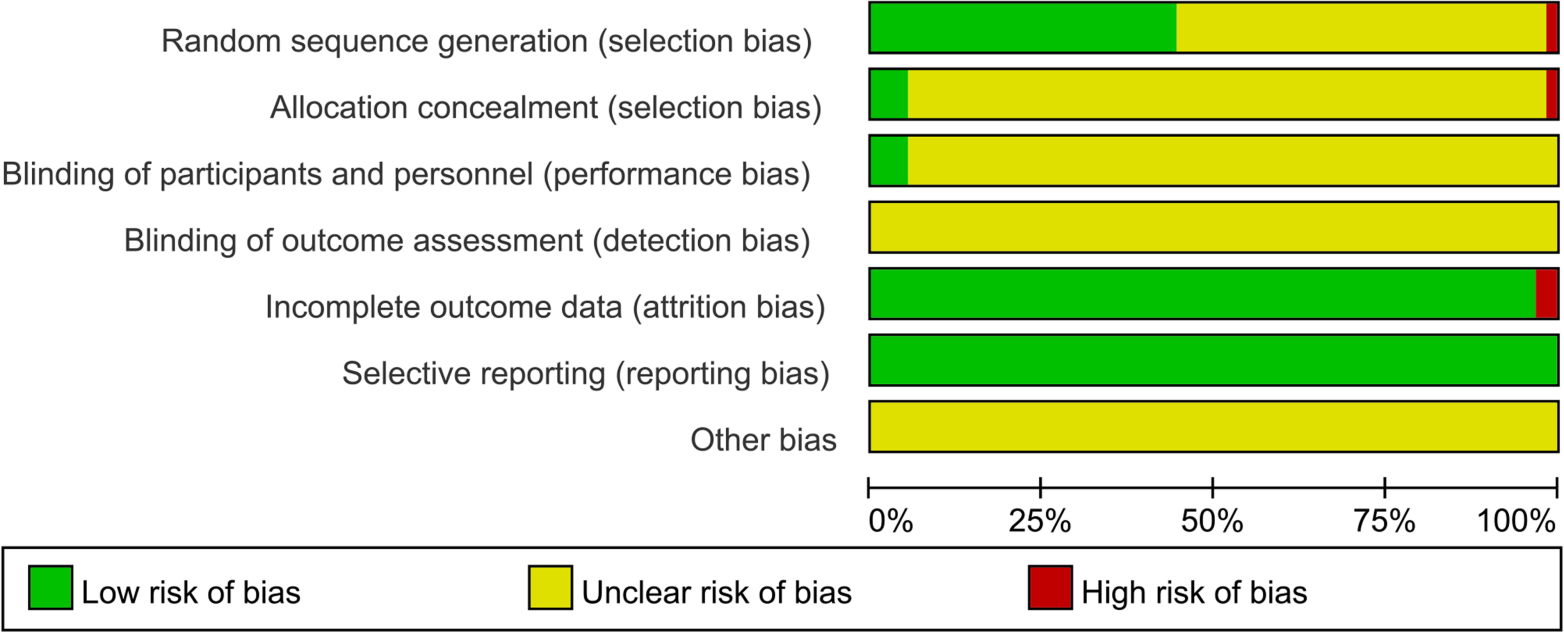
Risk of bias in these included trails.

The results of the quality assessment of the 59 selected papers are shown in Figure 2 and Supplementary Figure S1. Only 3 (13, 32, 58) of the 59 studies described the randomization method in details, 23 studies (27, 34, 38, 39, 46, 50, 51, 55, 60, 62–65, 67–69, 71–76, 78) used an appropriate randomization method (randomized numeric table method), and 1 study (66) used an inappropriate randomization method (dynamic randomized grouping and different medication administration); only 3 studies (13, 32, 58) described the allocation scheme concealment and double blinding; only 2 studies (58, 74) reported lost visits or missing cases (*n* = 21); all studies failed to selectively report outcome indicators or other biases. Twenty-six documents (13, 27, 32, 34, 38, 39, 46, 50, 51, 55, 58, 60, 62–65, 67–69, 71–76, 78) were rated as high quality (Jadad score ≥4) according to the modified Jadad scale.

### 3.3. Impact of QSYQ on outcome indicators

#### 3.3.1. Re-admission rates (RARs)

**Figure 3:**
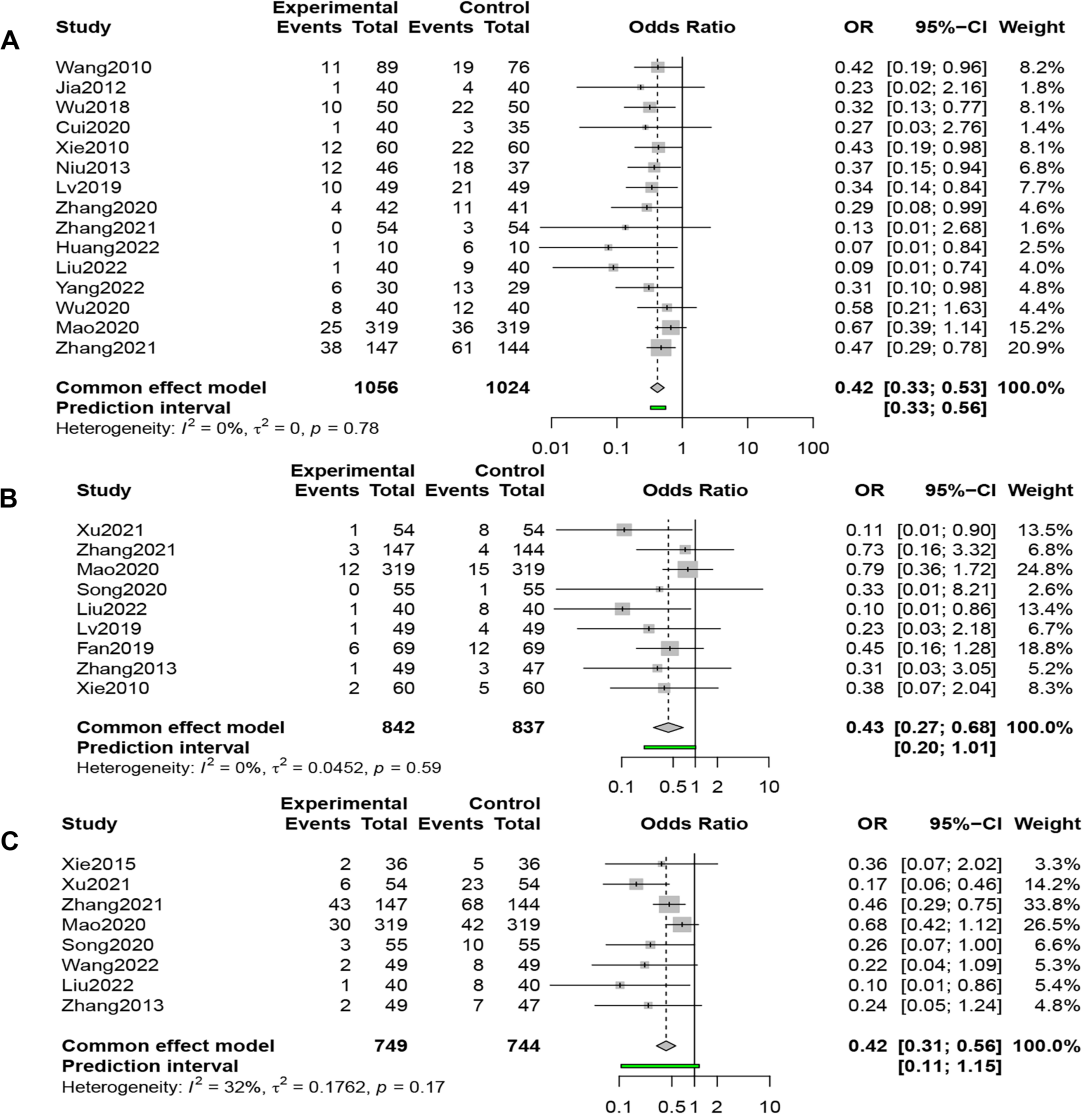
(**A**) Forest plot of readmission rate. (**B**) Forest plot of all-cause mortality. (**C**) Forest plot of MACE rate.

Fifteen studies (*n* = 2,080) compared RARs in the QSYQ group (*n* = 1,056) and the control group (*n* = 1,024); the results of the fixed-effects model (*I*^2 ^= 0%, *P* = 0.78 > 0.1) showed that QSYQ significantly reduced the RARs, [OR = 0.42, 95% CI (0.33, 0.53), *Z* = −7.26, *P* < 0.001] (Figure 3A).

#### 3.3.2. All-cause mortality(ACM)

Nine studies (*n* = 1,679) compared ACM in the QSYQ group (*n* = 842) and the control group (*n* = 837), and the results of the fixed-effects model (*I*^2 ^= 0%, *P* = 0.59 > 0.1) showed that QSYQ dramatically reduced the ACM, [OR = 0.43, 95% CI (0.27, 0.68), *Z* = −3.57, *P* < 0.001] (Figure 3B).

#### 3.3.3. Incidence of MACE

Eight studies (*n* = 1,493) compared the incidence of MACE in the QSYQ group (*n* = 749) and the control group (*n* = 744), and the results of the fixed-effects model (*I*^2 ^= 32%, *P* = 0.17 > 0.1) showed that QSYQ decreased the incidence of MACE significantly, [OR = 0.42, 95% CI (0.31, 0.56), *Z* = −5.82, *P* < 0.001] (Figure 3C).

#### 3.3.4. MLHFQ scores

**Figure 4:**
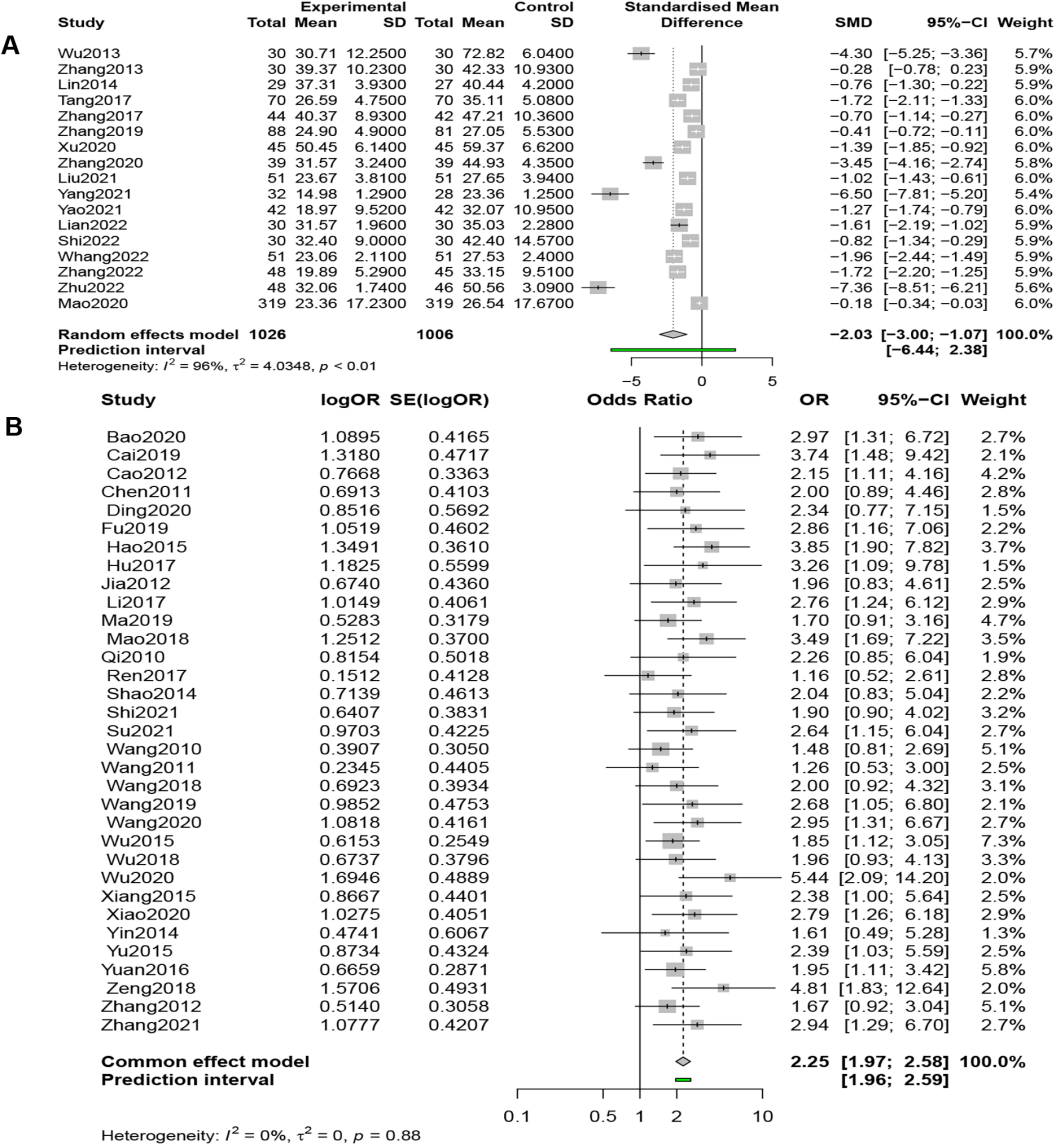
(**A**) Forest plot of MLHFQ scores. (**B**) Forest plot of clinical efficacy.

Seventeen studies (*n* = 2,032) compared the MLHFQ Scores, and the results of a random-effects model (*I*^2 ^= 96%, *P* < 0.01) demonstrated that combined QSYQ significantly improved patients' quality of life, [SMD = −2.03, 95% CI (−3.00, −1.07), *Z* = −4.12, *P* < 0.0001] (Figure 4A).

#### 3.3.5. Clinical efficacy rates (CERs)

Thirty-three studies (*n* = 3,289) compared CERs, and results from a fixed-effects model (*I*^2 ^= 0%, *P* = 0.879 > 0.1) showed that the QSYQ group was 2.25 times more likely to have an improvement of one grade or more in NYHA cardiac function classification than the control group, suggesting that the combined application of QSYQ was efficacious, [OR = 2.25, 95% CI (1.97, 2.58), *Z* = 11.77, *P* < 0.001] (Figure 4B).

#### 3.3.6. 6MWD

**Figure 5:**
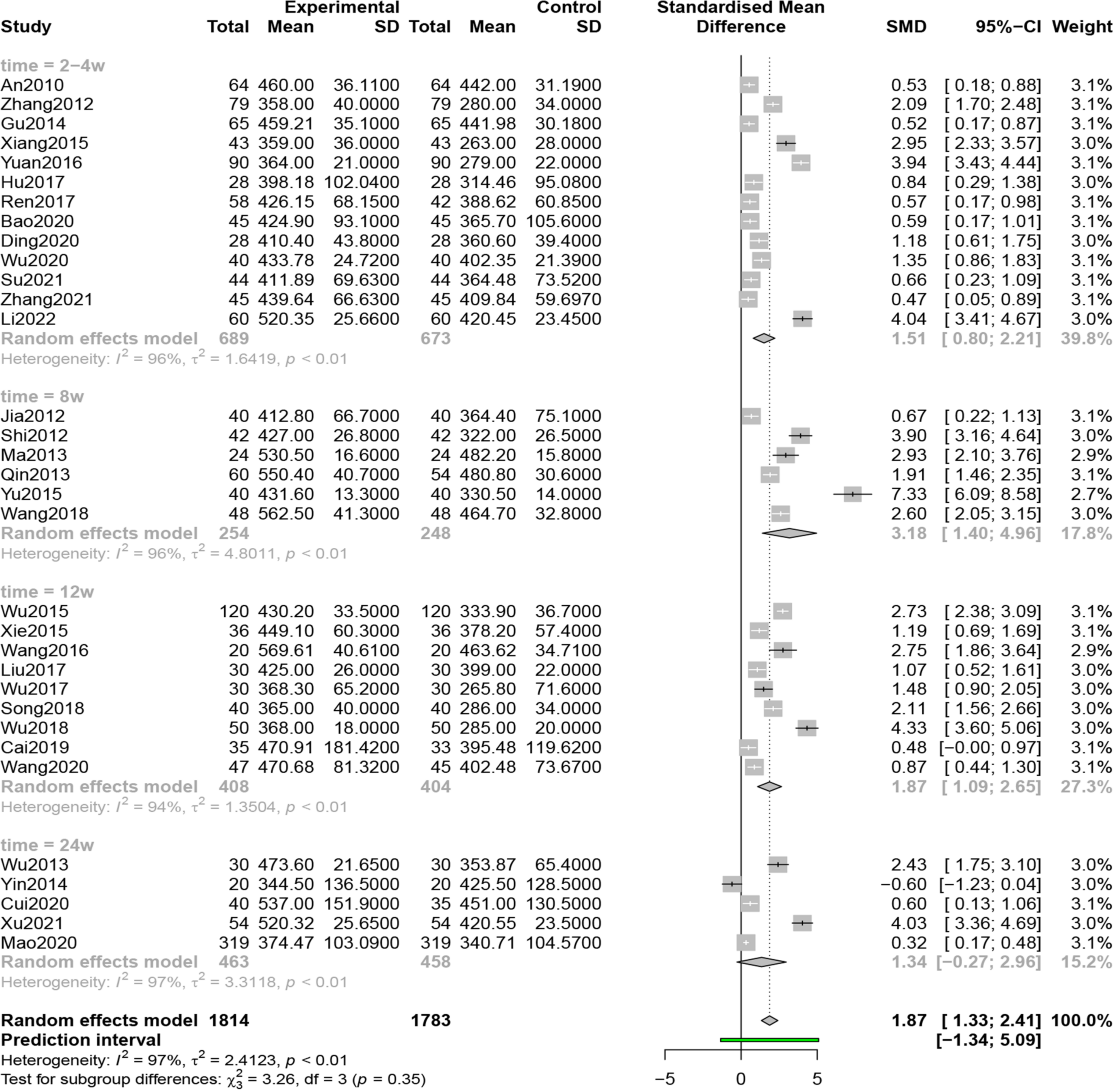
Forest plot of 6MWD.

Thirty-three studies (*n* = 3,597) reported 6MWD, and results from a random-effects model (*I*^2 ^= 97%, *P* < 0.01) showed that combined QSYQ markedly improved 6MWD, [SMD = 1.87, 95% CI (1.33, 2.41), *Z* = 6.81, *P* < 0.0001] (Figure 5).

#### 3.3.7. LVEF

**Figure 6:**
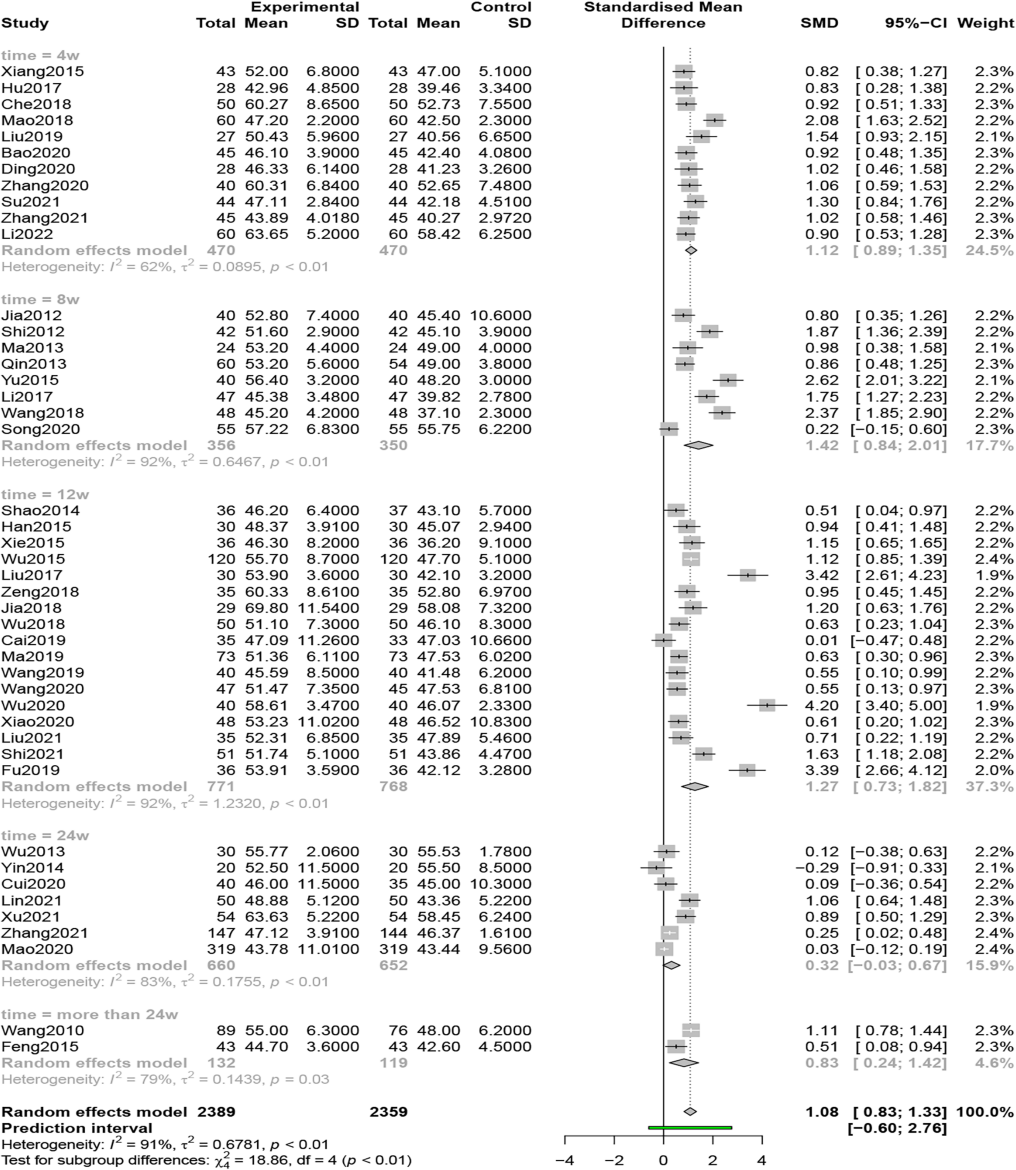
Forest plot of LVEF.

Forty-five studies (*n* = 4,748) compared LVEF, and the results of a random-effects model (*I*^2 ^= 91%, *P* < 0.01,) showed that QSYQ was able to significantly enhance LVEF, [SMD = 1.08, 95% CI (0.83, 1.33), *Z* = 8.44, *P* < 0.0001] (Figure 6).

#### 3.3.8. BNP

**Figure 7:**
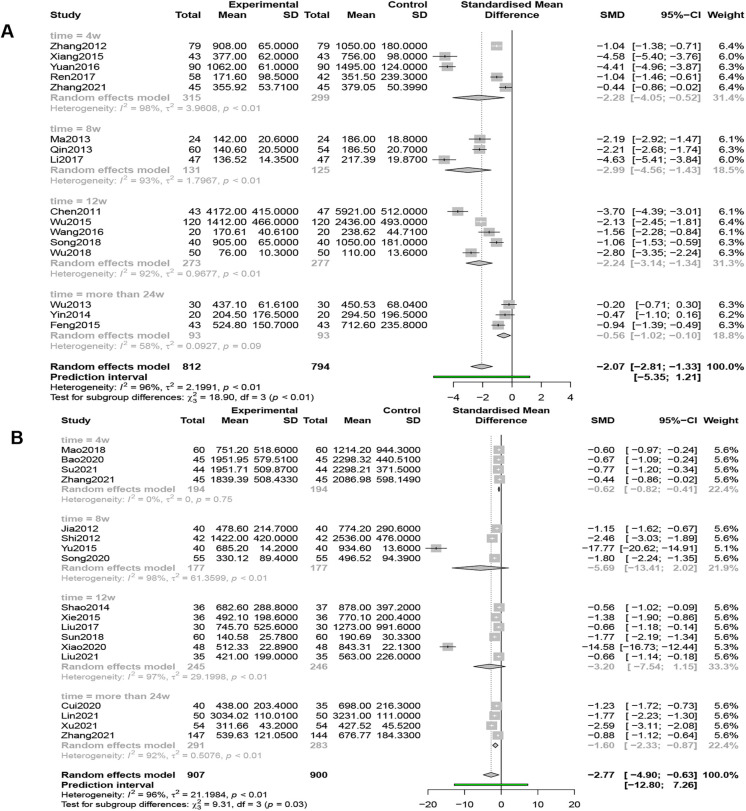
(**A**) Forest plot of BNP. (**B**) Forest plot of NT-ProBNP.

Sixteen studies (*n* = 1,606) compared BNP levels, and the results of a random-effects model (*I*^2 ^= 96%, *P* < 0.01) showed that QSYQ was able to significantly reduce BNP levels, [SMD = −2.07, 95% CI (−2.81, −1.33), *Z* = −5.48, *P* < 0.0001] (Figure 7A).

#### 3.3.9. NT-ProBNP

Eighteen studies (*n* = 1,807) reported NT-proBNP levels, and the results of a random-effects model (*I*^2 ^= 96%, *P* < 0.01) showed that QSYQ was able to significantly decrease the levels of NT-proBNP, [SMD = −2.77, 95% CI (−4.90, −0.63), *Z* = −2.54, *P* < 0.05] (Figure 7B).

#### 3.3.10. LVEDD

**Figure 8:**
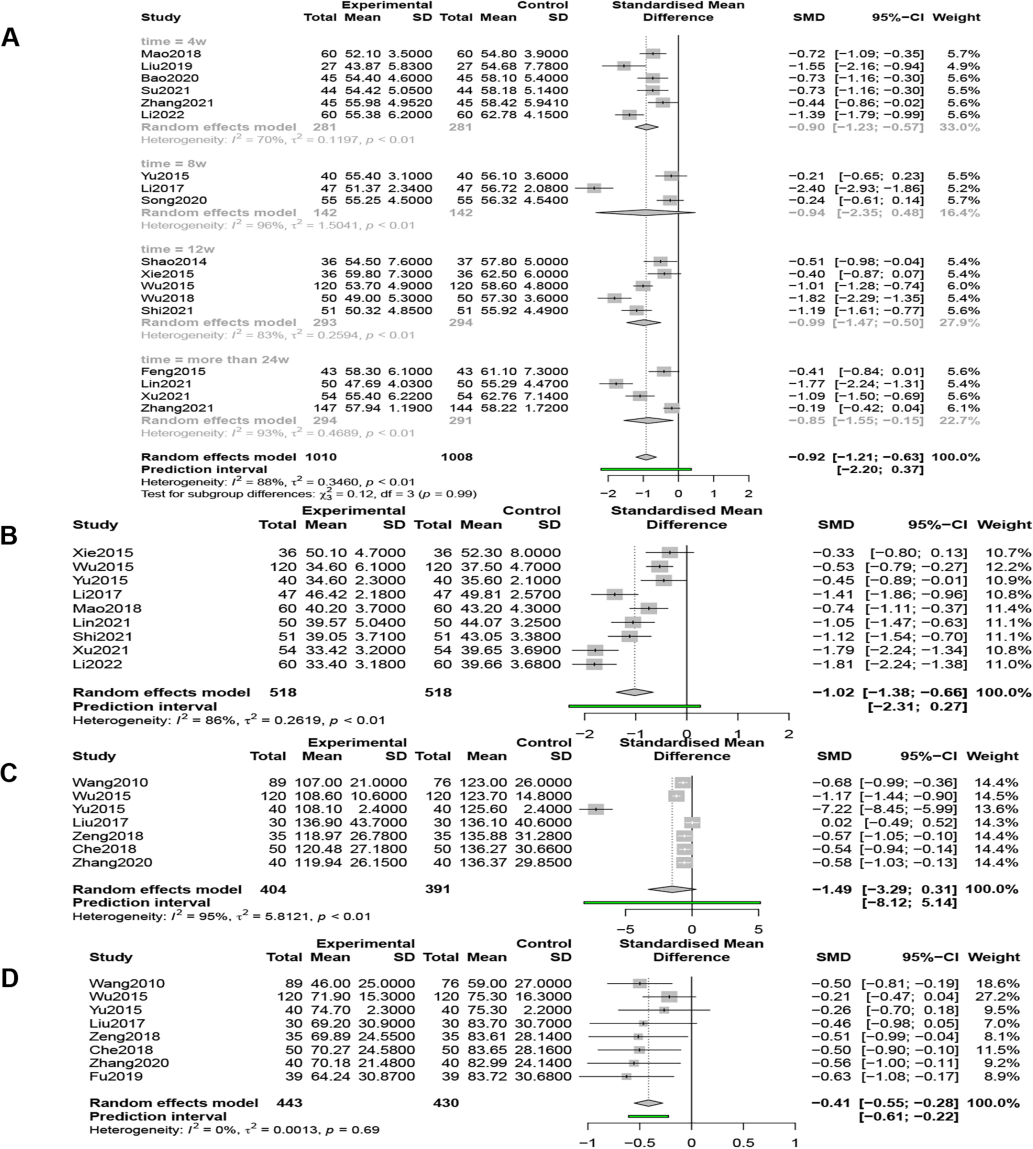
(**A**) Forest plot of LVEDD. (**B**) Forest plot of LVESD. (**C**) Forest plot of LVEDV. (**D**) Forest plot of LVESV.

Eighteen studies (*n* = 2,018) reported LVEDD, and a random-effects model (*I*^2 ^= 88%, *P* < 0.01) showed that QSYQ was able to significantly reduce LVEDD, [SMD = −0.92, 95% CI (−1.21, −0.63), *Z* = −6.21, *P* < 0.0001] (Figure 8A).

#### 3.3.11. LVESD

Nine studies (*n* = 1,036) reported LVESD, and a random-effects model (*I*^2 ^= 86%, *P* < 0.01) showed that QSYQ was able to significantly reduce LVESD, [SMD = −1.02, 95% CI (−1.38, −0.66), *Z* = −5.53, *P* < 0.0001] (Figure 8B).

#### 3.3.12. LVEDV

Seven studies (*n* = 795) compared LVEDV, and the results of the random effects model (*I*^2 ^= 95%, *P* < 0.01) showed that QSYQ was able to reduce LVEDV, but not statistically significant, [SMD = −1.49, 95% CI (−3.29, 0.31), *Z* = −1.62, *P* > 0.05] (Figure 8C).

#### 3.3.13. LVESV

Eight studies (*n* = 873) compared LVEDD, and the results of the fixed-effects model (*I*^2 ^= 0%, *P* = 0.69 > 0.01) showed that QSYQ was able to significantly reduce LVESV, [SMD = −0.41, 95% CI (−0.55, −0.28), *Z* = −5.93, *P* < 0.001] (Figure 8D).

#### 3.3.14. Safety

Adverse reactions were reported as an assessment indicator in 21 of 59 studies (*n* = 2,742), among which 13 studies mentioned that no adverse reactions occurred during the course of treatment in both QSYQ and control groups, and 8 studies reported in details about adverse reactions that occurred during the course of treatment such as hypotension, dizziness, headache and nausea. However, these reactions were common and relatively mild. None of the studies reported serious adverse effect that affected the course of the study, such as electrolyte disorders, severe hepatic and renal deficits, etc. Therefore, the addition of QSYQ did not cause significant or dramatic adverse events, and had a good safety and tolerability profile compared to the control group (Table 2).

**Table 2:** The side effects of included trails.

First author/year	QSYQ group	Control group	Adverse reactions in the QSYQ group
Cao 2012	0/64 (0%)	2/65 (3.08%)	NA
Jia 2012	0	0	NA
Zhang 2012	0	0	NA
Qin 2013	0	0	NA
Wu 2013	0	0	NA
Shao 2014	0	0	NA
Hao 2015	9/60 (15%)	24/60 (40%)	Hypotension, decreased heart rate
Xie 2015	0	0	NA
Xiang 2015	0	0	NA
Hu 2017	0	0	NA
Li 2017	0	0	NA
Ren 2017	0	0	NA
Wu 2017	0	0	NA
Wang 2018	5/48 (10.41%)	4/48 (8.33%)	Sinus bradycardia, hypotension, dry cough, gastrointestinal distress
Cai 2019	0	0	NA
Ma 2019	7/73 (9.59%)	6/73 (8.22%)	Hypotension, nausea, headache, electrolyte disturbances
Xiao 2020	3/48 (6.25%)	6/48 (12.5%)	Hypotension, dizziness, headache, nausea and vomiting
Zhang 2020	3/40 (7.5%)	2/40 (5.0%)	Hypotension, headache, nausea and vomiting
Shi 2021	4/51 (7.84%)	5/51 (9.80%)	Hypotension, dizziness, nausea
Zhang 2021	0	0	NA
Mao 2020	16/319 (5.01%)	18/319 (5.64%)	Cold, dizziness, nausea and vomiting, hematochezia, hyperkalemia, liver dysfunction

### 3.4. HFrEF

10, 3, 5, 8, 6, 13, 20, 6, 9 and 6 studies compared RARs, incidence of MACE, ACM, CERs, MLHFQ scores, 6MWD, LVEF, BNP, NT-ProBNP, and LVEDD, respectively, with respect to HFrEF, and the results were consistent with the above. [RARs: *I*^2 ^= 0%, OR = 0.43, 95% CI (0.32, 0.58), *P* < 0.01; incidence of MACE: *I*^2 ^= 38%, OR = 0.56, 95% CI (0.36, 0.89), *P* < 0.01; ACM: *I*^2 ^= 1%, OR = 0.48, 95% CI (0.28, 0.81), *P* < 0.01; CERs: *I*^2 ^= 0%, OR = 1.97, 95% CI (1.48, 2.61), *P* < 0.01; MLHFQ scores: *I*^2 ^= 98%, SMD = −2.49, 95% CI (−4.68, −0.29), *P* < 0.01; 6MWD: *I*^2 ^= 96%, SMD = 1.63, 95% CI (0.87, 2.39), *P* < 0.01; LVEF: *I*^2 ^= 93%, SMD = 1.03, 95% CI (0.66, 1.39), *P* < 0.01; BNP: *I*^2 ^= 93%, SMD = −1.28, 95% CI (−2.09, −0.48), *P* < 0.01; NT-ProBNP: *I*^2 ^= 84%, SMD = −1.29, 95% CI (−1.69, −0.88), *P* < 0.01; LVEDD: *I*^2 ^= 88%, SMD = −1.03, 95% CI (−1.21, −0.84), *P* < 0.01].

### 3.5. Prediction interval

Prediction intervals are not often reported but are more insightful and well suited to assess differences in intervention effects across settings (19). We observed that the prediction intervals and their respective 95% CIs in RARs, CERs, and LVESV almost overlapped, which may be related to the low heterogeneity of the studies. However, in other metrics, such as 6MWD, the combined effect size was 1.87 with a 95% CI (1.33, 2.41); yet, the prediction intervals ranged from −1.34 to 5.09 and contained values of zero or below zero. Similar results were observed in ACM (0.20, 1.01), incidence of MACE (0.11, 1.15), MLHFQ scores (−6.44, 2.38), LVEF (−0.60, 2.76), BNP (−5.35, 1.21), NT-ProBNP (−12.80, 7.26), LVEDD (−2.20, 0.37), LVESD (−2.31, 0.27), LVEDV (−8.12, 5.14). These suggest that QSYQ may not always be beneficial to patients in clinical practice, and may even be mildly harmful.

### 3.6. Subgroup analysis and meta-regression analysis

We performed subgroup analysis of 6MWD, LVEF, BNP, NT-proBNP, and LVEDD according to regimen, and in all subgroups the results were consistent with those described above. Although negative results were obtained in some subgroups (subgroup 24 weeks in 6MWD, subgroup 24 weeks in LVEF, subgroups 8 weeks and 12 weeks in NT-proBNP, and subgroup 8 weeks in LVEDD), there still showed a trend toward improvement. As the heterogeneity remained high, we performed meta-regression analysis of 6MWD and LVEF according to the quality of literature and mean age. The results showed that mean age (*p* = 0.0082 < 0.01) and literature quality (*p* = 0.0031 < 0.01) contributed to 24.49% of the heterogeneity (tau^2 ^= 1.7636, *R*^2 ^= 24.49%) for 6MWD, and, literature quality (*p* = 0.0007 < 0.001) contributed to 20.07% of the heterogeneity (tau^2 ^= 0.5364, *R*^2 ^= 20.07%) for LVEF. We therefore pooled high-quality literature and analyzed it by age and duration of treatment. We found a significant decrease in heterogeneity in the 60–65-year-old and 12 weeks-duration subgroups for 6MWD, and in the more-than-70-year-old and 2–4 weeks-duration subgroups for LVEF. Effect sizes did not change significantly across groups. There was no evidence that the quality of literature and mean age contributed to the heterogeneity of BNP, NT-proBNP, and LVEDD (*P* > 0.05) (Table 3).

**Table 3:** Meta-analysis results of high-quality studies.

Metrics	Subgroups	Studies	Participants	SMD (95% CI)	*I*^2^ (%)	P_Heterogeneity_
6MWD	Overall	13	1,637	1.33 (0.63, 2.03)	95	<0.01
Subgroup analysis by age	<60	4	359	2.45 (0.66, 4.25)	97	<0.01
	60–65	8	1,218	0.61 (0.42, 0.81)	57	0.02
	>65	1	60	2.43 (0.75, 3.10)	–	–
Subgroup analysis by treatment course	2–4 weeks	5	444	1.37 (0.07, 2.68)	96	<0.01
	8 weeks	1	80	0.67 (0.22, 1.13)	–	–
	12 weeks	3	232	0.84 (0.46, 1.23)	50	0.14
	24 weeks	4	881	1.82 (0.13, 3.52)	98	<0.01
LVEF	Overall	24	2,899	0.75 (0.56, 0.94)	86	<0.01
Subgroup analysis by age	<60	6	599	0.87 (0.45, 1.30)	81	<0.01
	60–65	12	1,604	0.70 (0.44, 0.95)	86	<0.01
	65–70	4	526	0.62 (−0.05, 1.29)	90	<0.01
	>70	2	170	1.01 (0.69, 1.33)	0	0.74
Subgroup analysis by treatment course	2–4 weeks	7	624	1.01 (0.84, 1.17)	0	0.9
	8 weeks	3	284	0.92 (0.04, 1.79)	92	<0.01
	12 weeks	8	719	0.75 (0.42, 1.08)	77	<0.01
	24 weeks	6	1,272	0.40 (0.04, 0.75)	85	<0.01

### 3.7. Sensitivity analysis

A sensitivity analysis was conducted to verify the stability and accuracy of the meta-analysis results. In the indicator LVEDV, the overall heterogeneity decreased (from 95% to 75%) after the document Yu 2015 was deleted, and the result of the combined effect size was reversed to be statistically significant (*P* < 0.01). We found that this document contributed to the largest heterogeneity through the Baujat plot. Among the other indicators, there was no significant change in heterogeneity and effect size, which suggests that the results of the meta-analysis were stable. See specific details at Supplementary Figure S2–S4.

### 3.8. Heterogeneity analysis

**Figure 9:**
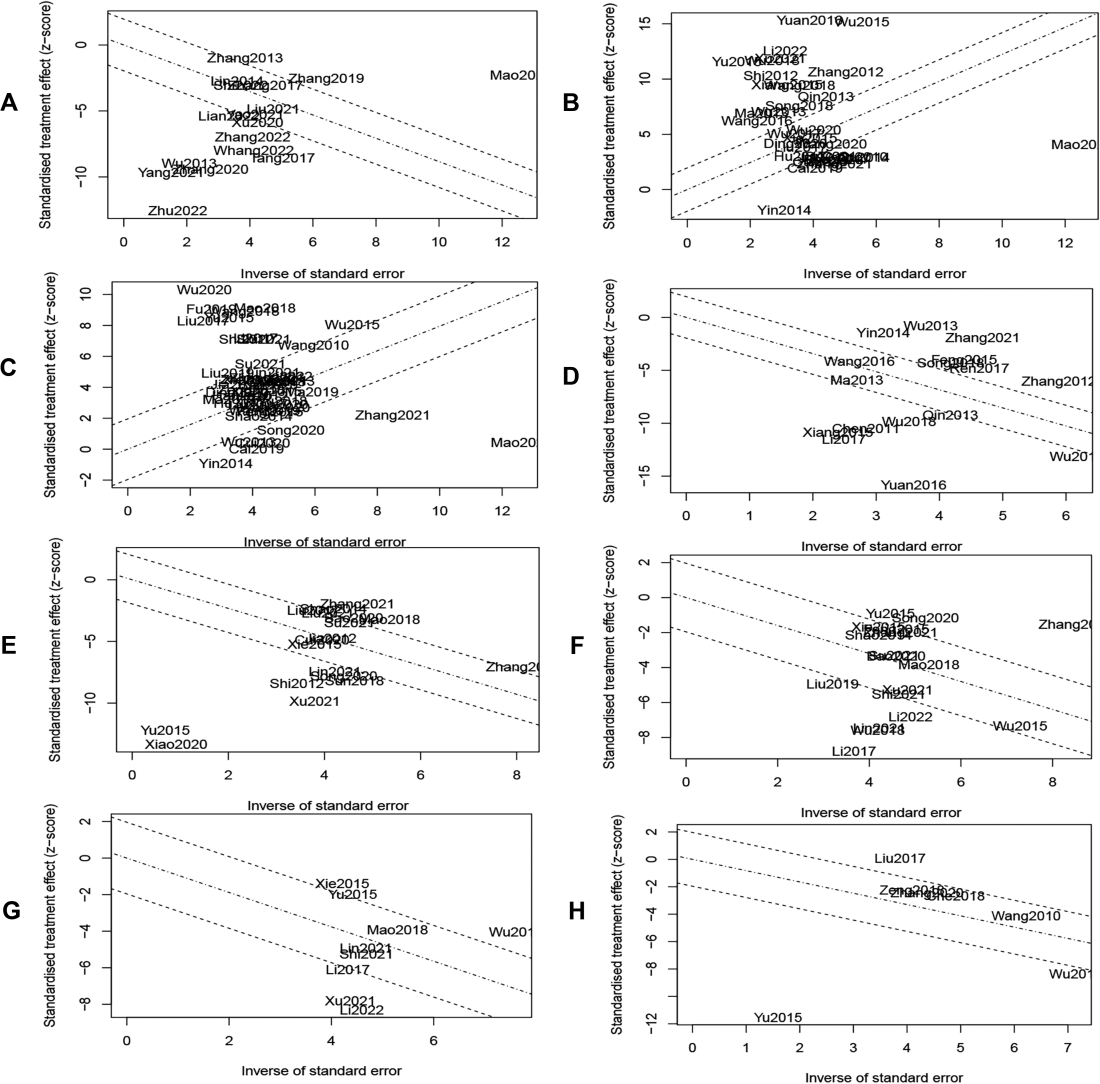
(**A**) MLHFQ scores. (**B**) 6MWD. (**C**) LVEF. (**D**) BNP. (**E**) NT-ProBNP. (**F**) LVEDD. (**G**) LVESD. (**H**) LVEDV.

The vast majority of subgroup analysis still had high heterogeneity, so we plotted Galbraith plots and Baujat plots (Figure 9 and Supplementary Figure S5). We found 11, 26, 16, 12, 9, 10, 5, and 2 studies to be the major sources of heterogeneity for MLHFQ scores, 6MWD, LVEF, BNP, NT-ProBNP, LVEDD, LVESD, and LVEDV, respectively. Heterogeneity was eliminated or significantly reduced by deleting the above outlier studies before re-performing the pooled analysis, but the combined effect sizes did not change significantly. [MLHFQ scores: SMD = −1.00, 95% CI (−1.19, −0.81), *I*^2 ^= 28%, P_heterogeneity _= 0.23; 6WMD: SMD = 1.12, 95% CI (0.93, 1.31), *I*^2 ^= 0%, P_heterogeneity_ = 0.57; LVEF: SMD = 0.89, 95% CI (0.81, 0.97), *I*^2 ^= 15%, P_heterogeneity _= 0.24; BNP: SMD = −2.10, 95% CI (−2.33, −1.86), *I*^2 ^= 0%, P_heterogeneity _= 0.48; NT-ProBNP: SMD = −0.87, 95% CI (−1.01, −0.73), *I*^2 ^= 29%, P_heterogeneity _= 0.19; LVEDD: SMD = −0.65, 95% CI (−0.80, −0.50), *I*^2 ^= 18%, P_heterogeneity _= 0.28; LVESD: SMD = −1.06, 95% CI (−1.33, −0.79), *I*^2 ^= 42%, P_heterogeneity _= 0.16; LVEDV: SMD = −0.53, 95% CI (−0.71, −0.34), *I*^2 ^= 27%, P_heterogeneity _= 0.24].

### 3.9. Publication bias

**Figure 10:**
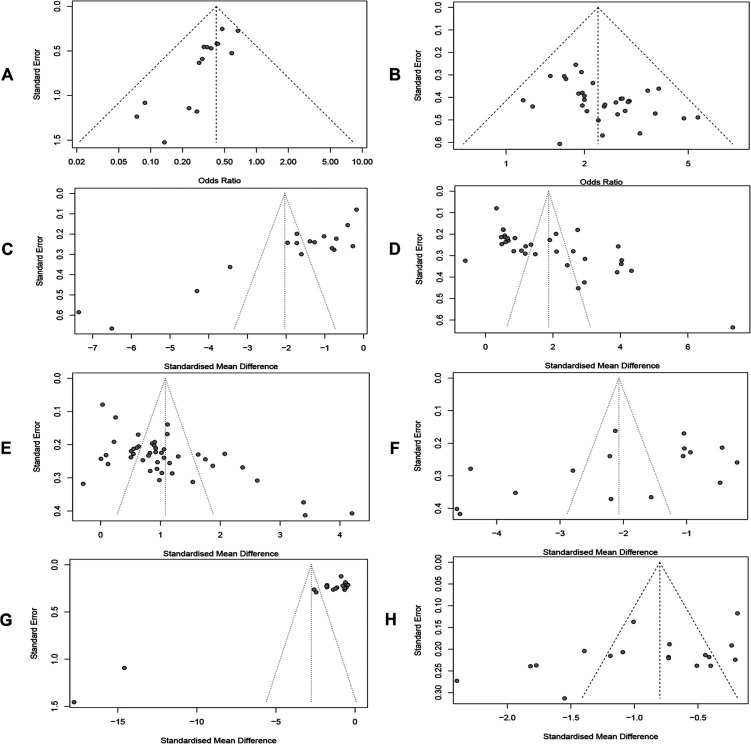
(**A**) Readmission rate. (**B**) Clinical efficacy. (**C**) MLHFQ score. (**D**) 6MWD. (**E**) LVEF. (**F**) BNP. (**G**) NT-proBNP. (**H**) LVEDD.

**Figure 11:**
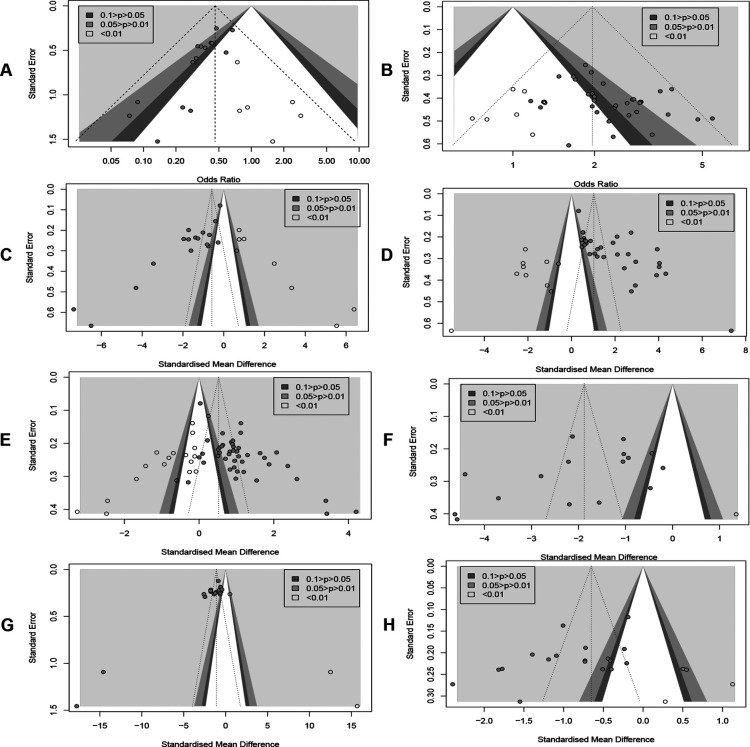
(**A**) Readmission rate. (**B**) Clinical efficacy. (**C**) MLHFQ score. (**D**) 6MWD. (**E**) LVEF. (**F**) BNP. (**G**) NT-ProBNP. (**H**) LVEDD.

Publication bias was detected by plotting funnel plots (Figure 10), which were visually asymmetric for RARs, CERs, MLHFQ scores, 6MWD, LVEF, NT-proBNP, and LVEDD, and the results of the Harbord test, Peters' test, or Egger's test provided corresponding support evidences. (RARs: Harbord test *P* < 0.01, Peters' test *P* < 0.01; CERs: Egger's test *P* = 0.0167 < 0.05; MLHFQ scores: Egger's test, *P* < 0.001; 6MWD: Egger's test, *P* < 0.001; LVEF: Egger's test, *P* < 0.001; NT-proBNP: Egger's test, *P* < 0.001; LVEDD: Egger's test, *P* = 0.0373 < 0.05). No significant publication bias was found in index BNP (Egger's test, *P* = 0.0676 > 0.05). The contour-enhanced funnel plots for the above 8 metrics were further trimmed and filled. The results (Figure 11) showed that 6 studies and 9 studies were added to the white area (not statistically significant, *P* > 0.1) in the funnel plots for RARs and CERs, respectively. In the funnel plots for MLHFQ scores, 6MWD, BNP, and NT-proBNP, 8, 9, 1, and 3 studies were added to the gray area, respectively. In the funnel plot for LVEF, 8 studies were added to the white area and 10 studies were added to the gray area, respectively. 1 study was added to the white area and 3 studies were added to the gray area in the funnel plot for LVEDD, respectively. In RARs, CERs, 6MWD, LVEF, LVEDD, and BNP, the effect sizes OR/SMD of recombination were not significantly altered after trimming and filling the funnel plot [RARs: OR = 0.46, 95% CI (0.37, 0.58), *P* < 0.001; CERs: OR = 1.97, 95% CI (1.74, 2.22), *P* < 0.001; 6MWD: SMD = 1.02, 95% CI (0.34, 1.71), *P* < 0.01; LVEF: SMD = 0.52, 95% CI (0.21, 0.84), *P* < 0.01; BNP: SMD = −1.88, 95% CI (−2.67, −1.08), *P* < 0.001; LVEDD: SMD = −0.65, 95% CI (−1.00, −0.30), *P* < 0.001]. However, there was a significant change in SMD of MLHFQ scores and NT-proBNP, which reversed to be statistically insignificant [MLHFQ scores: SMD = −0.58, 95% CI (−1.77, 0.60), *P* = 0.334; NT-proBNP: SMD = −1.10, 95% CI (−3.93, 1.74), *P* = 0.449].

### 3.10. GRADE assessment

By GRADE assessment, ACM, incidence of MACE, and LVESV were rated as moderate evidence, RARs and BNP were rated as low-quality evidence, while the rest of the indicators were rated as very low-quality evidence. Reasons for downgrading: (1) Regarding the risk of bias, only three papers described in details the implementation of randomization, allocation concealment, and blinding, whereas most of the studies just adopted appropriate randomization methods without specifying them. (2) In terms of inconsistency, a high degree of heterogeneity was found during the analysis, which were considered to be attributed to multiple factors such as study population, gender, disease duration, drug dispensing, and variable study quality. (3) Different degrees of publication bias were detected by drawing funnel plots (Table 4).

**Table 4:** GRADE-based assessment of evidence quality.

Quality assessment Importance							No. of patients			Effect	Quality	
No. of studies	Design	Risk of bias	Inconsistency	Indirectness	Imprecision	Other considerations	QSYQ + CT	CT	Relative (95% CI)	Absolute		
Rehospitalization												
15	Randomised trials	Serious^a^	No serious inconsistency	No serious indirectness	No serious imprecision	Reporting bias^b^	140/1,056 (13.3%)	260/1,042 (25%)	OR 0.42 (0.33–0.53)	127 fewer per 1,000 (from 100 fewer to 151 fewer)	⊕⊕○○ Low	Critical
All-cause mortality												
9	Randomised trials	Serious^a^	No serious inconsistency	No serious indirectness	No serious imprecision	None	27/842 (3.2%)	60/837 (7.2%)	OR 0.43 (0.27–0.68)	40 fewer per 1,000 (from 22 fewer to 51 fewer)	⊕⊕⊕○ Moderate	Critical
MACE												
8	Randomised trials	Serious^a^	No serious inconsistency	No serious indirectness	No serious imprecision	None	89/749 (11.9%)	171/744 (23%)	OR 0.42 (0.31–0.56)	118 fewer per 1,000 (from 87 fewer to 145 fewer)	⊕⊕⊕○ Moderate	Critical
MLHFQ (better indicated by lower values)												
17	Randomised trials	Serious^a^	Very serious^c^	No serious indirectness	No serious imprecision	Reporting bias^b^	1,026	1,006	–	SMD 2.03 lower (3.0–1.07 lower)	⊕○○○ Very low	Important
Clinical efficacy (better indicated by lower values)												
33	Randomised trials	Serious^a^	No serious inconsistency	No serious indirectness	No serious imprecision	Reporting bias^b^	1,657	1,632	–	OR 2.25 higher (1.97–2.58 higher)	⊕⊕○○ Low	Important
6MWD (better indicated by lower values)												
33	Randomised trials	Serious^a^	Very serious^c^	No serious indirectness	No serious imprecision	Reporting bias^b^	1,814	1,783	–	SMD 1.87 higher (1.33–2.41 higher)	⊕○○○ Very low	Important
LVEF (better indicated by lower values)												
45	Randomised trials	Serious^a^	Very serious^c^	No serious indirectness	No serious imprecision	Reporting bias^b^	2,389	2,359	–	SMD 1.08 higher (0.83–1.33 higher)	⊕○○○ Very low	Important
BNP (better indicated by lower values)												
16	Randomised trials	Serious^a^	Very serious^c^	No serious indirectness	No serious imprecision	None	812	794	–	SMD 2.07 lower (2.81–1.33 lower)	⊕○○○ Very low	Important
NT-ProBNP (better indicated by lower values)												
18	Randomised trials	Serious^a^	Very serious^c^	No serious indirectness	No serious imprecision	Reporting bias^b^	907	900	–	SMD 2.77 lower (4.90–0.63 lower)	⊕○○○ Very low	Important
LVEDD (better indicated by lower values)												
18	Randomised trials	Serious^a^	Very serious^c^	No serious indirectness	No serious imprecision	Reporting bias^b^	1,010	1,008	–	SMD 0.92 lower (1.21–0.63 lower)	⊕○○○ Very low	Important
LVESD (better indicated by lower values)												
9	Randomised trials	Serious^a^	Very serious^c^	No serious indirectness	No serious imprecision	None	518	518	–	SMD 1.02 lower (1.38–0.66 lower)	⊕○○○ Very low	Important
LVEDV (better indicated by lower values)												
7	Randomised trials	Serious^a^	Very serious^c^	No serious indirectness	Serious^d^	None	404	391	–	SMD 1.49 lower (3.29 lower–0.31 higher)	⊕○○○ Very low	Important
LVESV (better indicated by lower values)												
8	Randomised trials	Serious^a^	No serious inconsistency	No serious indirectness	No serious imprecision	None	443	430	–	SMD.41 lower (0.55–0.28 lower)	⊕⊕⊕○ Moderate	Important

^a^ There are large deviations in random allocation, allocation hiding, and blind design.

^b^ Asymmetric funnel plot showing publication bias.

^c^ The heterogeneity test *P* < 0.01, *I *^2^ > 75%.

^d^ The 95% confidence interval contains 0.

## 4. Discussion

To our knowledge, to date, this is the first and largest systematic evaluation and meta-analysis assessing the improvement of prognosis of patients with CIHF by the proprietary Chinese medicine QSYQ, and more comprehensive alternative metrics were pooled to evaluate its clinical efficacy and safety. The results of the meta-analysis showed that the combination of QSYQ with conventional Western medicine improved the prognosis of patients by reducing the RARs, the incidence of MACE, and ACM. This may be attributed to the improvement in cardiac function, exercise tolerance and quality of life, as well as the protective effect on cardiac structures. In terms of safety, no serious adverse events were reported in 21 studies comprising 2,742 patients, suggesting that QSYQ is relatively safe and well tolerated.

Despite the fact that standardized medications recommended by current international guidelines have been established as the cornerstone of treatment for HF and IHD, there is still a high residual risk in patients with CIHF (13). A large body of published evidence suggests that the coexistence of HF and coronary artery disease carries a high risk of adverse cardiac events and death, and that the risk of death increases progressively with the worsening of coronary artery disease (79); deterioration of patients' cardiac function, and socioeconomic deprivation lead to a worse quality of life, and a poor quality of life is strongly associated with recurrent readmissions and a higher mortality rate (80). Against this background, it is urgent to explore additional adjunctive therapies to mitigate this risk. The development of Traditional Chinese medicine (TCM) has provided more possibilities and options to improve the prognosis of CIHF patients (81). As a traditional Chinese medicine compound preparation, QSYQ has been widely used in China in the combined treatment of HF patients, owing to its efficacy of “benefiting qi and activating blood circulation”, and has achieved good therapeutic effects. A newly published reappraisal analysis of systematic reviews on QSYQ (82) points out the current lack of attention to the impact of QSYQ on mortality and readmission rates in patients with HF, even in one of the largest systematic evaluations incorporating 85 studies (11). With the popularization of the concept of heart failure vulnerable period (83) and the emphasis on the prognosis of patients with HF, more and more relevant studies have been published. Thus, we re-pooled and performed a meta-analysis with the prognostic index as the primary outcome indicator, and conducted a more adequate analysis of heterogeneity and a publication bias test.

The endpoint indicator is the real disease outcome, which is the event that patients are most concerned about and has the most immediate interests to them. The indicator can objectively reflect the real effect of the intervention, having great clinical significance and clinical reference value (84). Our results showed that QSYQ combined with conventional Western medications reduced RARs, incidence of MACE, and ACM in CIHF patients, and did not reveal significant heterogeneity, and the results remained stable even after being corrected for publication bias. In addition, we comprehensively summarized the alternative metrics that have been shown to be associated with poor prognosis in patients with CIHF, such as LVEF, which reliably reflects left heart function, 6MWD, which reflects the patient's exercise tolerance (85), as well as quantitative markers of HF, BNP and NT-ProBNP (86). Our results suggest that QSYQ adjunctive therapy for CIHF is favorable. However, it must be alarmed that a high degree of heterogeneity was observed in all of the above mentioned proxies.

Although subgroup and meta-regression analyses were performed, heterogeneity was not significantly eliminated. By looking at the Galbraith plots and the Baujat plots, we hypothesized that the heterogeneity between studies might involve multiple factors. On the one hand, the lack of study design may not only lead to differences in the evaluation of the intervention effect, but also create a higher risk of bias and lower the level of evidence in our study. On the other hand, the clinical heterogeneity could not always be further explored and addressed due to the lack of access to exhaustive clinical data from the original studies (11). For example, the patients' age, gender, disease duration, comorbidity characteristics, the dose and frequency of the specific drugs used, and the drug combinations or TCM dialectic typing, etc., we cannot rule out the interfering effect of these factors on the clinical efficacy. This limits to some extent the extrapolation of the results of this study. By trimming and filling the contour-enhanced funnel plots, we found that the cause of the funnel plot asymmetry may not be entirely attributable to publication bias. It is well known that systematic exaggeration of effect sizes resulting from small studies with poor study design can lead to funnel plot asymmetry and can introduce greater heterogeneity (87, 88). In addition, it is not uncommon for potential publication bias and heterogeneity to interact when both are coexisting (11, 89).

In addition, as can be seen from the wide prediction intervals, QSYQ may not always be beneficial and is even sometimes slightly detrimental in clinical applications. Just like conventional Western medicines, not every patient exhibits full tolerance, but we must also recognize the limitations of incorporating the principles of TCM. It is challenging to fully reconcile individualized treatment based on “one person, one prescription” and “dialectical treatment”, which are characteristics of TCM, with patient screening, which is centered on disease diagnosis (13). A clinical efficacy evaluation system guided by the combination of Western medicine diseases and TCM syndromes may provide an idea for future development (90, 91). In conclusion, we call for future RCTs to be pre-registered on relevant websites and to strictly follow the “CONSORT Extension for Chinese Herbal Medicine Formulas 2017” statement (92) for standardized study design. It is recommended to focus on the efficacy of QSYQ in patients with a specific HF type, such as HFpEF, or a certain TCM syndrome, such as qi deficiency and blood stasis, while negative findings and unfavorable results should not be concealed. In clinical practice, we need to follow our own rules of TCM development and integrate modern evidence-based medicine concepts, and develop a detailed and individualized dialectical medication plan according to the actual situation of patients based on the available evidence-based practice, rather than blindly applying it to all patients.

HF was categorized according to LVEF into heart failure with reduced ejection fraction (HFrEF, LVEF ≤40%), heart failure with mildly reduced ejection fraction (HFmrEF, LVEF 41%–49%) and heart failure with preserved ejection fraction (HFpEF, LVEF ≥50%) (15). Several RCTs have found that patients with HFmrEF are similar to HFrEF in terms of treatment benefit (15), and both have similar pathophysiologic characteristics. Based on this, patients with HFmrEF were also included in this study. Analysis of HFrEF showed that treatment method that combines QSYQ with conventional Western medicines improved its prognosis and improved all indicators. However, we did not perform a subgroup analysis of HFpEF due to the lack of available clinical data. Although HFpEF and HFrEF have similar symptoms and signs, HFpEF has not benefited from conventional drug treatment (93, 94). Due to the heterogeneity and complexity of the pathogenesis and comorbidities, no substantial breakthroughs have been made in its pathogenesis and treatment options, and its continued prevalence and poor prognosis should not be underestimated (95). Current studies point to multiple mechanisms of systemic inflammatory response and its induced endothelial dysfunction, oxidative stress, abnormal cardiac energy metabolism, and microvascular dysfunction that lead to increased myocardial fibrosis, myocardial remodeling, and diastolic dysfunction (95). A study innovatively found that epicardial adipose tissue (EAT) promotes myocardial inflammation by activating inflammatory vesicle-mediated cellular pyroptosis in adipocytes and constructs an EAT-myocardium axis, which provides a new strategy and a new way of thinking for the treatment of HFpEF (94). Previous studies have pointed out that astragalus with Salvia miltiorrhiza is a core drug for the treatment of HFpEF because it can regulate oxidative stress and glycolipid metabolism through multi-components and multi-targets (96). Similarly, a meta-analysis showed that QSYQ improves cardiac function and exercise tolerance in patients with HFpEF (10). This suggests that although QSYQ is beneficial in treating HFpEF, it does not provide the most direct support for its effect in improving prognosis. Further studies with large-scale, multicenter RCTs are still needed in the future.

In addition, diabetes mellitus is one of the leading causes of HF, and the mortality is significantly increased when HF is complicated by diabetes mellitus. They are independent risk factors for each other (97). Reactive oxygen species (ROS)-mediated oxidative stress, glucose-lipid metabolism disorders caused by insulin resistance, perfusion insufficiency due to endothelial dysfunction, autonomic dysfunction, and activation of multiple inflammatory responses may be potential mechanisms of diabetic heart failure (98, 99). In the inflammatory response, NOD-like receptor protein 3 (NLRP3) inflammatory vesicles activated by multiple pathways, such as high-glucose and high-fat stimuli, oxidative stress, endoplasmic reticulum stress, and calcium overload, induce the secretion of a large number of pro-inflammatory cytokines through the cascade of inflammation, which then mediate the process of cellular pyroptosis and promote myocardial injury and fibrosis (94, 100). Several studies have shown that QSYQ ameliorates myocardial injury by inhibiting excessive autophagy and NLRP3 inflammatory vesicles (101) and protects cardiomyocytes from high glucose-induced injury (97). Also, it can promote the repair of diabetic myocardial ischemic injury by up-regulating the levels of Sirt1 and eNOS, increasing NO bioavailability, preserving endothelial function, improving neovascularization, and inhibiting myocardial fibrosis and myocardial apoptosis (102). This shows that the proprietary Chinese medicine QSYQ has great therapeutic potential. More notably, compared with the traditional hypoglycemic effect of empagliflozin (EMP), its prognostic improvement and cardioprotective effect on HF patients are more compelling (98). Therefore, it will be interesting and valuable to investigate whether the combination of EMP with QSYQ can bring more therapeutic opportunities and greater benefits for patients with diabetes and HF.

Over the past decade or so, several studies have been conducted in an attempt to elucidate the underlying mechanisms by which QSYQ improves IHD. In a rat model of HF constructed by coronary artery ligation, it was found that QSYQ had a significant myocardial protective effect on HF rats, which may improve the degree of myocardial fibrosis by inhibiting the TGF-*β*1/Smads pathway, and decrease myocardial cell apoptosis by inhibiting the caspase-3 signaling pathway (103). The results of a network pharmacology showed that the active ingredients in QSYQ, such as astragaloside, Salvianic acid A, and ginsenoside Rg1, could synergistically regulate the targets in the HIF-1 signaling pathway to inhibit the expression of this signaling pathway and protect cardiomyocytes (104). It has also been suggested that QSYQ may inhibit the oxidative damage of myocardial tissues in HF model rats by activating the Nrf2/HO-1 signaling pathway, and thus exert its protective effect on cardiomyocyte damage (105). In summary, the above preclinical findings support to some extent the protective and ameliorative effects of QSYQ on CIHF, which are realized through multiple targets and pathways.

In short, the tremendous advantages of TCM in synergistic treatment of HF have attracted more and more attention from researchers. Moreover, research on TCM has evolved from the original macro syndrome differentiation and treatment to elucidating its role and mechanism from multiple dimensions, such as molecular biology and metabolomics. Individualized precision therapy for HF guided by evidence-based medicine evidence is becoming an objective and universally accepted model of care in treatment protocols. However, standardized and scientific TCM clinical efficacy evaluation system and high-quality clinical trials are still expected to provide solid support for TCM to prevent and treat HF in order to increase the contribution of TCM.

We must acknowledge the limitations of this study:(1) Although we systematically assessed the effect of QSYQ on prognosis and clinical symptoms in CIHF for the first time, the overall quality of the included studies was low and most of them did not use placebo controls, which somewhat compromised the level of evidence and affected the reliability of our results; (2) The lack of available specific data did not allow us to further analyze heterogeneity and publication bias or to assess differences in the efficacy of QSYQ in specific populations by further subgroup analysis. (3) HFpEF subtypes were not analyzed. (4)The observation time of most studies was limited to less than 1 year, so rigorously designed large-sample clinical trials with long-term follow-up are still needed to further evaluate its efficacy.

## 5. Conclusions

The available evidence suggests that the combined application of QSYQ can further improve CIHF patients' cardiac function, exercise tolerance, and quality of life, alleviate clinical symptoms, and ultimately improve their prognosis with a favorable safety profile. However, limited by the quality and high heterogeneity of the literature, we must be more conservative and cautious about the present results and approach QSYQ dialectically. We look forward to the implementation of rigorously designed and high-quality RCTs to further refine our conclusions.

## Data Availability

The original contributions presented in the study are included in the article/**Supplementary Material**, further inquiries can be directed to the corresponding authors.
