# Supplementary material for: Clinical efficacy and safety of adjunctive treatment of chronic ischemic heart failure with Qishen Yiqi dropping pills: a systematic review and meta-analysis
Source: Front Cardiovasc Med. 2023 Dec 18;10:1271608. doi: 10.3389/fcvm.2023.1271608 (PMC10765592; doi:10.3389/fcvm.2023.1271608)

Supplementary Material

# Supplementary Data

## Search strategies:

①Search Strategy of CNKI(n=375):

（主题：心力衰竭(精确））OR（主题：心衰（精确））OR（主题：心功能不全（精确））OR（主题：心脏衰竭（精确））OR（主题：心功能衰竭（精确））OR（主题：心脏功能衰竭（精确））OR（主题：心衰竭（精确））OR （主题：慢性心衰（精确））OR（主题：慢性心力衰竭（精确））OR（主题：慢性心功能不全（精确））AND（（主题：芪参益气滴丸（精确））OR（主题：芪参益气(精确）））；

②Search Strategy of Wanfang(n=679):

主题:(心力衰竭 or 心衰 or 心功能不全 or 心脏衰竭 or 心功能衰竭 or 心脏功能衰竭 or 心衰竭 or 慢性心衰 or 慢性心力衰竭 or 慢性心功能不全) and 主题:(芪参益气滴丸 or 芪参益气) ；

③Search Strategy of VIP(n=321):

题名或关键词=心力衰竭+cardiac failure+heart failure+慢性心衰竭+慢性心功能不全+慢性心力衰竭+急性充血性心力衰竭+慢性心脏功能衰竭+急性心衰+慢性充血性心衰+慢性心力衰竭+充血性心衰+充血心力衰竭+慢性心力衰+慢性心功能衰竭+充血性心力衰竭+慢性心衰+性心力衰竭+急性心功能衰竭+心功能不全+心衰+慢性充血性心力衰竭+急性心力衰竭+心脏衰竭+心脏功能衰竭+心功能衰竭+心衰竭AND 題名或关键词=芪参益气滴丸+芪参益气；

④Search Strategy of CBM(n=266):

| 7 | (#6) AND (#3) | 266 |
| --- | --- | --- |
| 6 | (#5) OR (#4) | 614 |
| 5 | "芪参益气"[常用字段:智能] | 614 |
| 4 | "芪参益气滴丸"[不加权:扩展] | 536 |
| 3 | (#2) OR (#1) | 143928 |
| 2 | "心衰"[常用字段:智能] OR "心功能不全"[常用字段:智能] OR "心脏衰竭"[常用字段:智能] OR "心功能衰竭"[常用字段:智能] OR "心脏功能衰竭"[常用字段:智能] OR "心衰竭"[常用字段:智能] OR "慢性心衰"[常用字段:智能] OR "慢性心力衰竭"[常用字段:智能] OR "慢性心功能不全"[常用字段:智能] | 143928 |
| 1 | "心力衰竭"[不加权:扩展] | 104192 |

⑤Search Strategy of PubMed(n=19):

(("Heart Failure"[Mesh]) OR ((heart failure[Title/Abstract]) OR (Cardiac Failure[Title/Abstract]) OR (Heart Decompensation[Title/Abstract]) OR (Decompensation, Heart[Title/Abstract]) OR (Heart Failure, Right-Sided[Title/Abstract]) OR (Heart Failure, Right Sided[Title/Abstract]) OR (Right-Sided Heart Failure[Title/Abstract]) OR (Right Sided Heart Failure[Title/Abstract]) OR (Myocardial Failure[Title/Abstract]) OR (Congestive Heart Failure[Title/Abstract]) OR (Heart Failure, Congestive[Title/Abstract]) OR (Heart Failure, Left-Sided[Title/Abstract]) OR (Heart Failure, Left Sided[Title/Abstract]) OR (Left-Sided Heart Failure[Title/Abstract]) OR (Left Sided Heart Failur[Title/Abstract]))) AND ((((((Qishen Yiqi Dripping Pills[Title/Abstract]) OR (Qishen Yiqi Dripping Pill[Title/Abstract])) OR (Qishen Yiqi DropPill[Title/Abstract])) OR (Qishen Yiqi[Title/Abstract])) OR (Qishen Yiqi droplet[Title/Abstract])) OR (Qishen Yiqi Pills[Title/Abstract]))；

⑥Search Strategy of Web of Science（n=38）:

| #1 | Heart failure (Title) or Cardiac Failure (Title) or Heart Decompensation (Title) or Decompensation, Heart (Title) or Heart Failure, Right-Sided (Title) or Heart Failure, Right Sided (Title) or Right-Sided Heart Failure (Title) or Right Sided Heart Failure (Title) or Myocardial Failure (Title) or Congestive Heart Failure (Title) or Heart Failure, Congestive (Title) or Heart Failure, Left-Sided (Title) or Heart Failure, Left Sided (Title) or Left-Sided Heart Failure (Title) or Left Sided Heart Failure (Title) | 669397 |
| --- | --- | --- |
| #2 | Qishen Yiqi Dripping Pills (Title) or Qishen Yiqi Dripping Pill (Title) or Qishen Yiqi DropPill (Title) or Qishen Yiqi (Title) or Qishen Yiqi droplet (Title) or Qishen Yiqi Pills (Title) | 133 |
| #3 | #1 and #2 | 38 |

⑦Search Strategy of Embase（n=24）:

| #5 | #3 AND #4 | 24 |
| --- | --- | --- |
| #4 | qishen yiqi dripping pills':ab,ti OR 'qishen yiqi dripping pill':ab,ti OR 'qishen yiqi droppill':ab,ti OR 'qishen yiqi':ab,ti OR 'qishen yiqi droplet':ab,ti OR 'qishen yiqi pills':ab,ti | 69 |
| #3 | #1 OR #2 | 717337 |
| #2 | 'heart failure':ab,ti OR‘cardiac failure':ab,ti OR 'heart decompensation':ab,ti OR 'decompensation, heart':ab,ti OR 'heart failure, right-sided':ab,ti OR 'heart failure, right sided':ab,ti OR 'right-sided heart failure':ab,ti OR 'right sided heart failure':ab,ti OR 'myocardial failure':ab,ti OR 'congestive heart failure':ab,ti OR 'heart failure, congestive':ab,ti OR 'heart failure, left-sided':ab,ti OR 'heart failure, left sided':ab,ti OR 'left-sided heart failure':ab,ti OR 'left sided heart failure':ab,ti | 358197 |
| #1 | 'heart failure'/exp OR 'heart failure' | 711918 |

⑧Search strategies in the cochrane library(n=8)

| #1 | MeSH descriptor: [Heart Failure] explode all trees | 14340 |
| --- | --- | --- |
| #2 | (Cardiac Failure):ti,ab,kw or (Heart Decompensation):ti,ab,kw or (Decompensation, Heart):ti,ab,kw or (Heart Failure, Right-Sided):ti,ab,kw or (Heart Failure, Right Sided):ti,ab,kw or (Right-Sided Heart Failure):ti,ab,kw or (Right Sided Heart Failure):ti,ab,kw or (Myocardial Failure):ti,ab,kw or (Congestive Heart Failure):ti,ab,kw or (Heart Failure, Congestive):ti,ab,kw or (Heart Failure, Left-Sided):ti,ab,kw or (Heart Failure, Left Sided):ti,ab,kw or (Left-Sided Heart Failure):ti,ab,kw or (Left Sided Heart Failure):ti,ab,kw | 27423 |
| #3 | #1 OR #2 | 33748 |
| #4 | (Qishen Yiqi Dripping Pills):ti,ab,kw or (Qishen Yiqi Dripping Pill ):ti,ab,kw or (Qishen Yiqi DropPill):ti,ab,kw or (Qishen Yiqi ):ti,ab,kw or (Qishen Yiqi droplet ):ti,ab,kw or (Qishen Yiqi Pills):ti,ab,kw | 26 |
| #5 | #3 AND #4 | 8 |

## Characteristics of the studies included in the meta-analysis

Table S1 Characteristics of the studies included in the meta-analysis

| **First author /Year** | **Region** | **Language** | **Numbers**  **QSYQ/Control** | **Age (years)**  **QSYQ/Control** | **Sex**  **Male/Female** | **NYHA II/III/IV**  **QSYQ group** | **NYHA II/III/IV**  **Control group** | **LVEF** | **Intervention**  **measures** | **Treatment course (weeks)** | **Outcomes** | **Jadad score** |
| --- | --- | --- | --- | --- | --- | --- | --- | --- | --- | --- | --- | --- |
| An YX 2010 | Henan | Chinese | 128（64/64） | 67±4  /68±6 | Q：36/28；  C：37/27 | 0/51/13 | 0/53/11 | NA | Q(0.5g,TID)+CT/CT | 2 | ⑥ | 2 |
| Qi JX 2010 | Tianjin | Chinese | 60（30/30） | 74.97±9.49  /75.20±7.37 | Q：15/15；  C：16/14 | NA | NA | NA | Q(0.5g,TID)+CT/CT | 4 | ⑤ | 2 |
| Wang D 2010 | Liaoning | Chinese | 165（89/76） | 57.8 | 91/74 | NA | NA | ≤45% | Q(0.5g,TID)+CT/CT | 48 | ①/⑤/⑦/⑫/⑬ | 2 |
| Chen SR 2011 | Sichuan | Chinese | 90（43/47） | 58.8 | 43/47 | NA | NA | NA | Q(0.5g,TID)+CT/CT | 12 | ⑤/⑧ | 2 |
| WangWD2011 | Heilongjiang | Chinese | 80（40/40） | 65.7 | 38/42 | NA | NA | ≤45% | Q(0.5g,TID)+CT/CT | 2 | ⑤ | 2 |
| Cao LF 2012 | Henan | Chinese | 129（64/65） | 61±8  /61±8 | Q：36/28；  C：35/30 | 31/33/0 | 25/40/0 | NA | Q(0.5g,TID)+CT/CT | 6 | ⑤ | 2 |
| Jia HL 2012 | Henan | Chinese | 80（40/40） | 62. 4±8. 6 | 36/44 | NA | NA | ≤40% | Q(0.5g,TID)+CT/CT | 8 | ①/⑤/⑥/⑦/⑨ | 4 |
| Shi YC 2012 | Jiangsu | Chinese | 84（42/42） | 67.5±6.7  /66.5±8.2 | Q：22/20；  C：23/19 | NA | NA | ≤40% | Q(0.5g,TID)+CT/CT | 8 | ⑥/⑦/⑨ | 2 |
| Zhang J 2012 | Guangdong | Chinese | 158（79/79） | 60±5.5  /62±2.5 | Q：40/39；  C：42/37 | 0/42/37 | 0/38/41 | NA | Q(0.5g,TID)+CT/CT | 4 | ⑤/⑥/⑧ | 2 |
| Ma AL 2013 | Jiangsu | Chinese | 48（24/24） | NA | NA | NA | NA | NA | Q(0.5g,TID)+CT/CT | 8 | ⑥/⑦/⑧ | 2 |
| Qin CH 2013 | Henan | Chinese | 114（60/54） | 69.5±10.6  /68.7±9.8 | Q：37/23；  C：32/22 | 14/30/16 | 14/26/14 | ≤45% | Q(0.5g,TID)+CT/CT | 8 | ⑥/⑦/⑧ | 2 |
| Wu TC 2013 | Guizhou | Chinese | 60（30/30） | 66.12±6.63  /65.00±7.96 | Q：17/13；  C：16/14 | 22/8/0 | 24/6/0 | ≤45% | Q(0.5g,TID)+CT/CT+  Placebo | 24 | ④/⑥/⑦/⑧ | 7 |
| Gu MF 2014 | Jiangsu | Chinese | 130（65/65） | 66.8±3.9  /67.8±5.6 | Q：43/22；  C：38/27 | 0/53/12 | 0/58/7 | NA | Q(0.5g,TID)+CT/CT | 2 | ⑥ | 2 |
| Shao B 2014 | Shanghai | Chinese | 73（36/37） | 68.5±9.6  /66.7±11.2 | Q：22/14；  C：24/13 | 8/19/9 | 10/16/11 | NA | Q(0.5g,TID)+CT/CT | 12 | ⑤/⑦/⑨/⑩ | 4 |
| Yin W 2014 | Henan | Chinese | 40（20/20） | NA | Q：11/9；  C：10/10 | 8/7/5 | 9/7/4 | ≤45% | Q(0.5g,TID)+CT/CT | 24 | ⑤/⑥/⑦/⑧ | 2 |
| Feng GP 2015 | Henan | Chinese | 86（43/43） | 59.9±6.7  /61.2±5.8 | Q：28/15；  C：28/15 | NA | NA | ≤40% | Q(0.5g,TID)+CT/CT | 72 | ⑦/⑧/⑩ | 2 |
| Han XJ 2015 | Tianjin | Chinese | 60（30/30） | 60.5±10.4  /66.2±9.6 | Q：18/12；  C：17/13 | 8/16/6 | 9/15/6 | ≤50% | Q(0.5g,TID)+CT/CT | 12 | ⑦ | 2 |
| Hao HQ 2015 | Henan | Chinese | 120（60/60） | 68.40±1.85  /65.85±1.70 | Q：34/26；  C：34/26 | NA | NA | NA | Q(0.5g,TID)+CT/CT | 4 | ⑤ | 4 |
| Xie F 2015 | Shǎnxi | Chinese | 72（36/36） | 65.2±7.5  /63.0±8.3 | Q：23/13；  C：20/16 | NA | NA | ≤50% | Q(0.5g,TID)+CT/CT | 12 | ③/⑦/⑥/⑨/⑩/⑪ | 4 |
| Wu TK 2015 | Henan | Chinese | 240（120/120） | 70±7.1  /70±6.1 | Q：70/50；  C：68/52 | 10/64/46 | 12/56/52 | NA | Q(0.5g,TID)+CT/CT | 12 | ⑤/⑥/⑦/⑧/⑩/⑪/⑫/⑬ | 2 |
| Xiang SZ 2015 | Hubei | Chinese | 86（43/43） | 60.0±7.4  /62.0±6.5 | Q：23/20；  C：22/21 | 0/30/13 | 0/33/11 | NA | Q(0.5g,TID)+CT/CT | 4 | ⑤/⑥/⑦/⑧ | 2 |
| Yu CY 2015 | Zhejiang | Chinese | 80（40/40） | 60.5±2.0  /60.1±1.1 | Q：24/16；  C：23/17 | 5/20/15 | 6/22/12 | NA | Q(0.5g,TID)+CT/CT | 8 | ⑤/⑥/⑦/⑨/⑩/⑪/⑫/⑬ | 2 |
| Wang LZ 2016 | Jiangsu | Chinese | 40（20/20） | 55.12±4.45  /56.58±4.29 | Q：9/11；  C：10/10 | NA | NA | NA | Q(0.5g,TID)+CT/CT | 12 | ⑥/⑧ | 2 |
| Yuan L 2016 | Henan | Chinese | 180（90/90） | 62±5.8  /64±5.2 | Q：47/43；  C：42/48 | NA | NA | NA | Q(0.5g,TID)+CT/CT | 4 | ⑤/⑥/⑧ | 2 |
| Hu J 2017 | Sichuan | Chinese | 56（28/28） | 67.46±3.25  /67.00±4.41 | Q：17/11；  C：15/13 | NA | NA | ≤40% | Q(0.5g,TID)+CT/CT | 4 | ⑤/⑥/⑦ | 2 |
| Li P 2017 | Shǎnxi | Chinese | 94（47/47） | 58.36±5.39  /59.14±4.97 | Q：29 /18；  C：27 /20 | 0/31/16 | 0/32/15 | NA | Q(0.5g,TID)+CT/CT | 8 | ⑤/⑦/⑧/⑩/⑪ | 4 |
| Liu YY 2017 | Jiangsu | Chinese | 60（30/30） | 63.89±7.23  /61.49±7.75 | Q：16 /14；  C：17 /13 | NA | NA | ≤45% | Q(0.5g,TID)+CT/CT | 12 | ⑥⑦/⑨/⑫/⑬ | 2 |
| Ren LF 2017 | Neimenggu | Chinese | 100（58/42） | 70.2 | 68/32 | NA | NA | NA | Q(0.5g,TID)+CT/CT | 4 | ⑤/⑥/⑧ | 2 |
| Wu p 2017 | Sichuan | Chinese | 60(30/30) | 61.5±2.3  /61.8±2.1 | Q：16 /14；  C：20 /10 | NA | NA | NA | Q(0.5g,TID)+CT/CT | 12 | ⑥ | 2 |
| Zeng J 2018 | Shǎnxi | Chinese | 70（35/35） | 78.54±6.25  /78.56±6.23 | Q：19 /16；  C：20 /15 | NA | NA | NA | Q(10pills,TID)+[clopidogrel](javascript:;)/[clopidogrel](javascript:;) | 12 | ⑤/⑦/⑫/⑬/ | 4 |
| Che QF 2018 | Heilongjiang | Chinese | 100（50/50） | 61.43±10.66  /64.12±11.34 | Q：27 /23；  C：29/21 | 15/16/19 | 13/17/20 | NA | Q(0.5g,TID)+CT/CT | 4 | ⑦/⑫/⑬/ | 4 |
| Jia YQ 2018 | Tianjin | Chinese | 58（29/29） | 63.44±6.20  /63.21±6.54 | Q：12 /17；  C：16/13 | NA | NA | NA | Q(10pills,TID)+[clopidogrel](javascript:;)/[clopidogrel](javascript:;) | 12 | ⑦ | 2 |
| Mao BY 2018 | Henan | Chinese | 120（60/60） | 68.2±9.7  /68.1±9.4 | Q：35/25；  C：34/26 | 16/31/13 | 17/30/13 | ≤45% | Q(0.5g,TID)+CT/CT | 4 | ⑤/⑦/⑨/⑩/⑪ | 2 |
| Song GF 2018 | Heilongjiang | Chinese | 80（40/40） | 62.4±8.6 | 39/41 | NA | NA | ≤40% | Q(0.5g,TID)+CT/CT | 12 | ⑥/⑧ | 2 |
| Sun LX 2018 | Hebei | Chinese | 120（60/60） | 61.29±2.35  /60.23±2.77 | Q：36/24；  C：35/25 | 0/60/0 | 0/60/0 | ≤45% | Q(0.5g,TID)+CT/CT | 12 | ⑨ | 4 |
| Wang QD 2018 | Guangdong | Chinese | 96（48/48） | 64.2±6.1  /65.3±6.7 | Q：25/23；  C：27/21 | NA | NA | ≤45% | Q(0.5g,TID)+CT/CT | 8 | ⑤/⑥/⑦ | 2 |
| Wu BL 2018 | Shānxi | Chinese | 100（50/50） | 65±5.6  /64±4.8 | Q：24/26；  C：25/25 | 10/35/5 | 8/36/6 | ≤40% | Q(0.5g,TID)+CT/CT | 12 | ①/⑤/⑥/⑦/⑧/⑩ | 2 |
| Cai XD 2019 | Henan | Chinese | 80（40/40） | 61.8±7.44  /64.52±6.96 | Q：25/10；  C：23/10 | 17/18/0 | 22/11/0 | NA | Q(0.5g,TID)+CT/CT+  Placebo | 12 | ⑤/⑥/⑦ | 7 |
| Liu YG 2019 | Hebei | Chinese | 54（27/27） | 64.77±6.49  /63.53±7.63 | Q：15/12；  C：14/13 | NA | NA | ≤45% | Q（0.5g,TID)+CT /CT | 4 | ⑦/⑩ | 2 |
| Ma TF 2019 | Henan | Chinese | 146（73/73） | 55.69±4.87  /54.51±4.36 | Q：36/37；  C：38/35 | 39/34/0 | 40/33/0 | NA | Q(0.52g,TID)+CT/CT | 12 | ⑤/⑦ | 4 |
| Wang W 2019 | Shandong | Chinese | 80（40/40） | 69.5±9.8  /68.8±9.6 | Q：24/16；  C：23/17 | NA | NA | NA | Q(0.5g,TID)+CT/CT | 12 | ⑤/⑦ | 2 |
| Bao LN 2020 | Neimenggu | Chinese | 90（45/45） | 62.3±8.21  /63.0±8.82 | Q：23/22；  C：25/20 | 25/13/7 | 23/14/8 | NA | Q(0.5g,TID)+CT/CT | 4 | ⑤/⑥/⑦/⑨/⑩ | 4 |
| Cui ZS 2020 | Beijing | Chinese | 75（40/35） | 55.16±6.02  /52.48±5.26 | Q：28/12；  C：22/13 | 10/25/5 | 6/22/7 | ≤50% | Q(0.5g,TID)+CT/CT | 24 | ①/⑥/⑦/⑨ | 4 |
| Ding SG 2020 | Anhui | Chinese | 56（28/28） | 59.5±9.8  /58.8±9.6 | Q：11/17；  C：12/16 | NA | NA | NA | Q(0.5g,TID)+CT/CT | 4 | ⑤/⑥/⑦ | 4 |
| Wang YN 2020 | Henan | Chinese | 92（47/45） | 63.04±11.87  /61.78±10.04 | Q：27/20；  C：27/18 | NA | NA | NA | Q(0.5g,TID)+CT/CT | 12 | ⑤/⑥/⑦ | 4 |
| Wu DM 2020 | Jiangsu | Chinese | 80（40/40） | 67.68±3.87  /66.51±4.66 | Q：22/18；  C：24/16 | NA | NA | NA | Q(0.5g,TID)+CT/CT | 12 | ⑤/⑥/⑦ | 1 |
| Xiao P 2020 | Guangdong | Chinese | 96（48/48） | 60.82±3.19  /60.52±3.33 | Q：28/20；  C：26/22 | 23/22/3 | 21/23/4 | NA | Q(0.5g,TID)+CT/CT | 12 | ⑤/⑦/⑨ | 4 |
| Zhang HW2020 | Henan | Chinese | 80（40/40） | 63.09±6.36  /63.15±6.46 | Q：22/18；  C：23/17 | 12/12/16 | 11/12/17 | ≤45% | Q(0.52g,TID)+CT/CT | 4 | ⑦/⑫/⑬ | 4 |
| Lin F 2021 | Liaoning | Chinese | 100（50/50） | 84.00±13.67  /87.97±7.65 | Q：32/18；  C：28/22 | NA | NA | ≤40% | Q(0.5g,TID)+CT/CT | 24 | ⑦/⑨/⑩/⑪ | 4 |
| Liu Q 2021 | Liaoning | Chinese | 70（35/35） | 70.7±9.5  /69.8±8.6 | Q：22/13；  C：23/12 | NA | NA | ≤50% | Q(0.5g,TID)+CT/CT | 12 | ⑦/⑨ | 2 |
| Shi Y 2021 | Henan | Chinese | 102（51/51） | 67.89±5.02  /69.02±4.56 | Q：27/24；  C：29/22 | 15/25/11 | 17/24/10 | NA | Q(0.52g,TID)+CT/CT | 12 | ⑤/⑦/⑩/⑪ | 4 |
| Su JN 2021 | Neimenggu | Chinese | 88（44/44） | 61.36±6.04  /61.09±6.74 | Q：23/21；  C：25/19 | 17/21/6 | 20/19/5 | NA | Q(0.5g,TID)+CT/CT | 4 | ⑤/⑥/⑦/⑨/⑩ | 4 |
| Xu Y 2021 | Beijing | Chinese | 110（55/55） | 54.7±5.3  /55.4±5.4 | Q：34/20；  C：33/21 | NA | NA | NA | Q(0.5g,TID)+CT/CT | 24 | ②/③/⑥/⑦/⑨⑩/⑪ | 4 |
| Zhang KX 2021 | Hebei | Chinese | 300（150/150） | 67.89±9.97  /67.02±9.39 | Q：96/54；  C：99/51 | 30/73/47 | 29/77/44 | NA | Q(0.5g,TID)+CT/CT | 48 | 1. /②/③/⑦/⑨/⑩ | 4 |
| Zhang RX 2021 | Neimenggu | Chinese | 90（45/45） | 62.69±8.415  /65.04±9.283 | Q：26/19；  C：24/21 | 20/17/8 | 18/18/9 | NA | Q(0.5g,TID)+CT/CT | 4 | ⑤/⑥/⑦/⑧/⑨/⑩ | 4 |
| Li QR 2022 | Qinghai | Chinese | 120（60/60） | 55.70±6.28  /55.65±6.25 | Q：34/26；  C：33/27 | NA | NA | NA | Q(0.5g,TID)+CT/CT | 4 | ⑥/⑦/⑩/⑪/ | 4 |
| Mao JY 2020 | Multi-center | English | 640(320/320） | 65.0±9.1  /64.9±8.9 | Q：233/96；  C：238/81 | 149/149/21 | 133/167/19 | ≤45% | Q(0.52g,TID)+CT/CT+Placebo | 24 | ①/②/③/④/⑥/⑦ | 7 |
| Fu B 2019 | Henan | Chinese | 72（36/36） | 55.24±5.27  /56.18±4.53 | Q：19/17；  C：21/15 | NA | NA | NA | Q(0.5g,TID)+CT/CT | 12 | ⑤/⑦/⑬ | 2 |
| Song SY 2020 | Shanghai | Chinese | 110（55/55） | 63.6±9.2  /64. 8±8. 9 | Q：31/24；  C：30/25 | NA | NA | ＞45% | Q(0.5g,TID)+CT/CT | 8 | 1. /⑦/⑨/⑩ | 4 |

Notes: Q/QSYQ： Qishen Yiqi dropping pills; CT： conventional treatment；①Re-admission rates(RARs); ②[All-Cause Mortality](javascript:;)（ACM）；③Incidence of MACE: Major Adverse Cardiovascular Events；④MLHFQ scores；⑤Clinical Efficacy Rate(CERs)；⑥6MWD:6-minute walking distance; ⑦LVEF: left ventricular ejection fraction；⑧BNP: brain natriuretic peptide；⑨NT-pro BNP: N-terminal prohormone of BNP；⑩LVEDD: left ventricular end-diastolic dimensions；⑪LVESD: left ventricular end-systolic dimensions； ⑫LVEDV: left ventricular end-diastolic volume；⑬LVESV: Left ventricular end-systolic volume.

# Supplementary Figures

## Risk of bias summary.

Supplementary Figure S1.


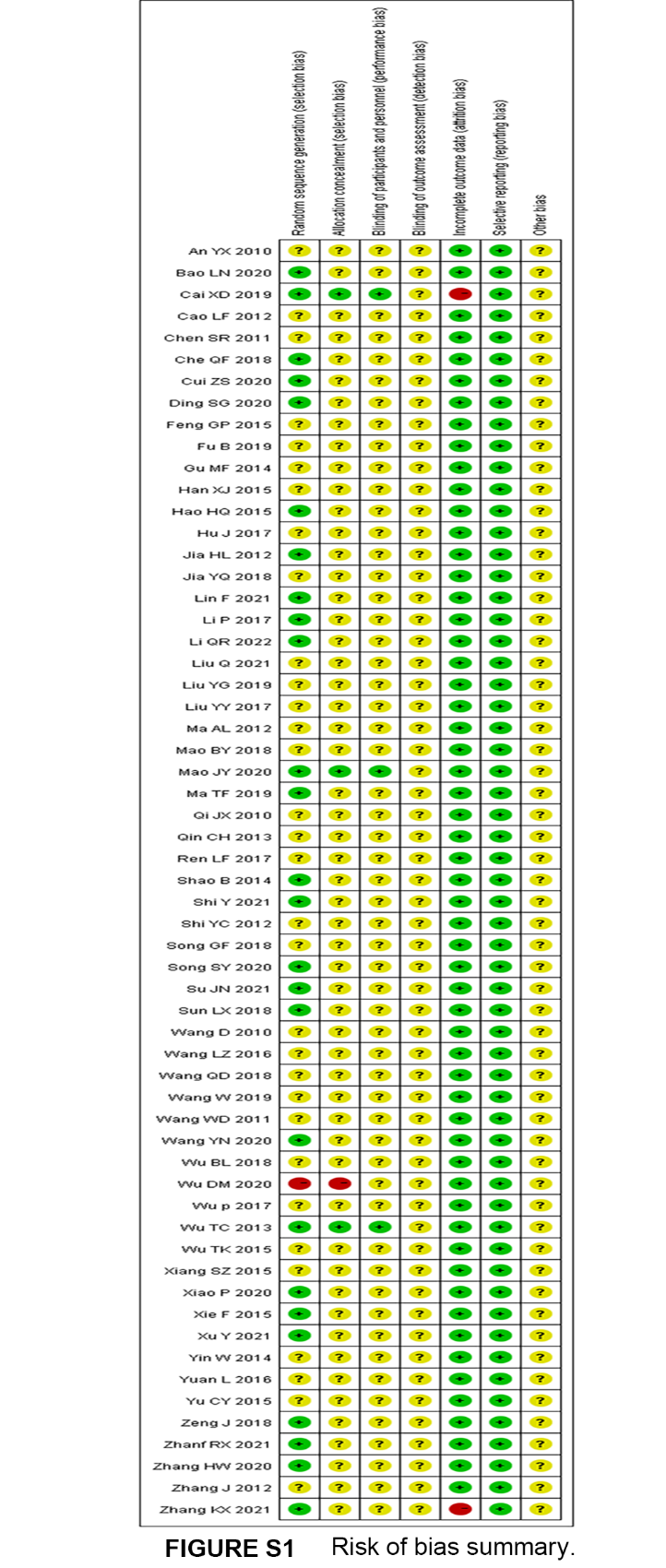


## Sensitivity analyses:

Supplementary Figure S2.


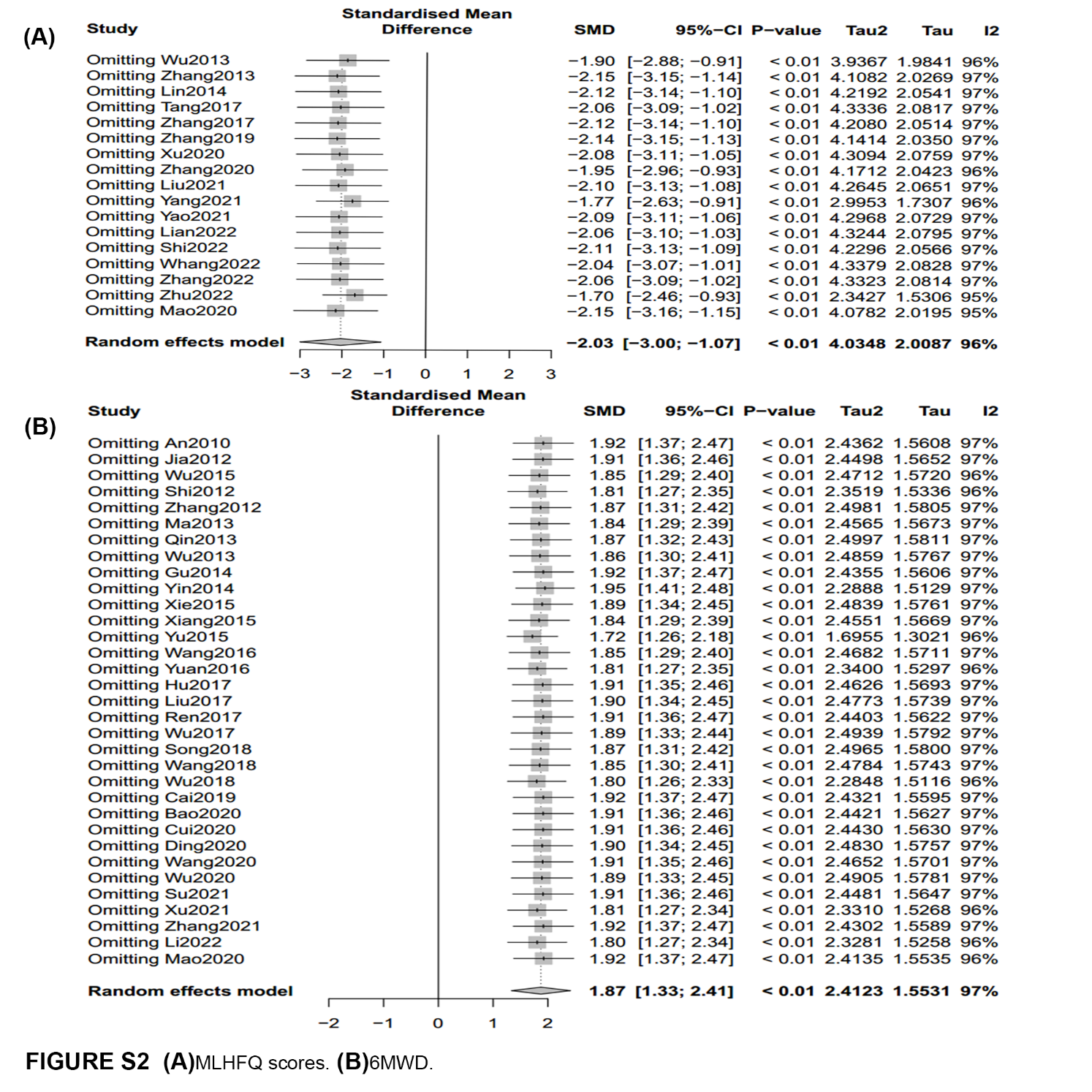


Supplementary Figure S3.


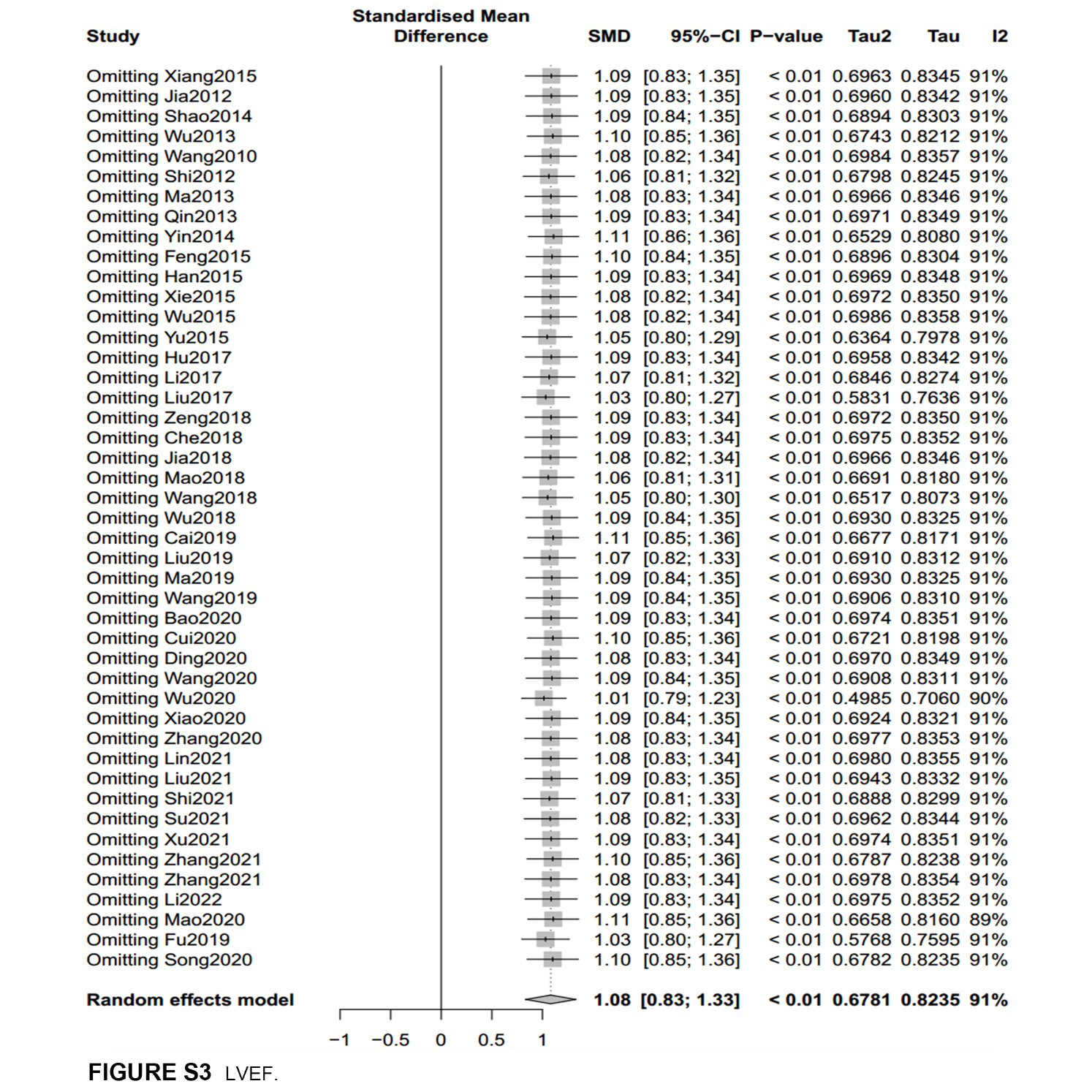


Supplementary Figure S4.


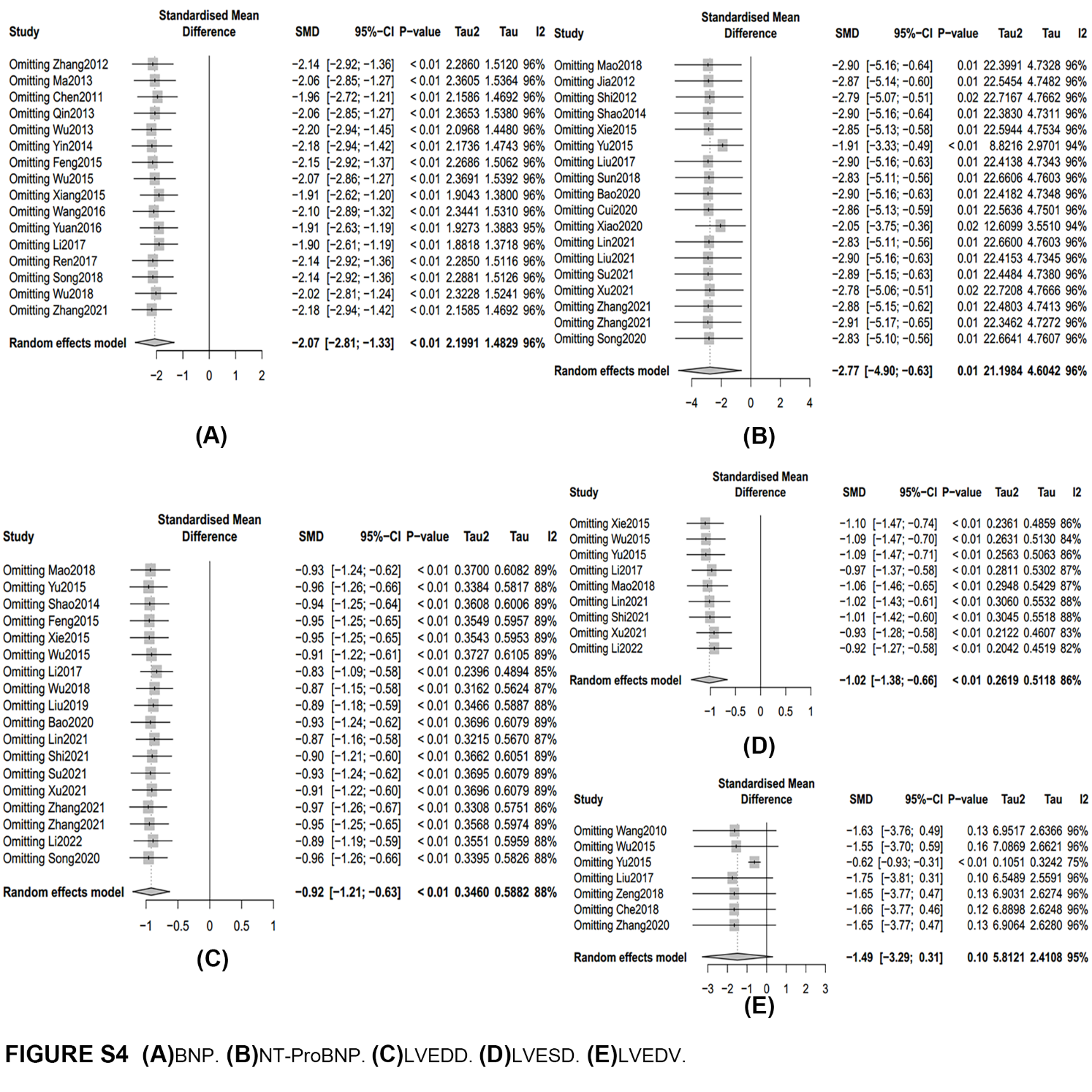


## Bajat plots:

Supplementary Figure S5.


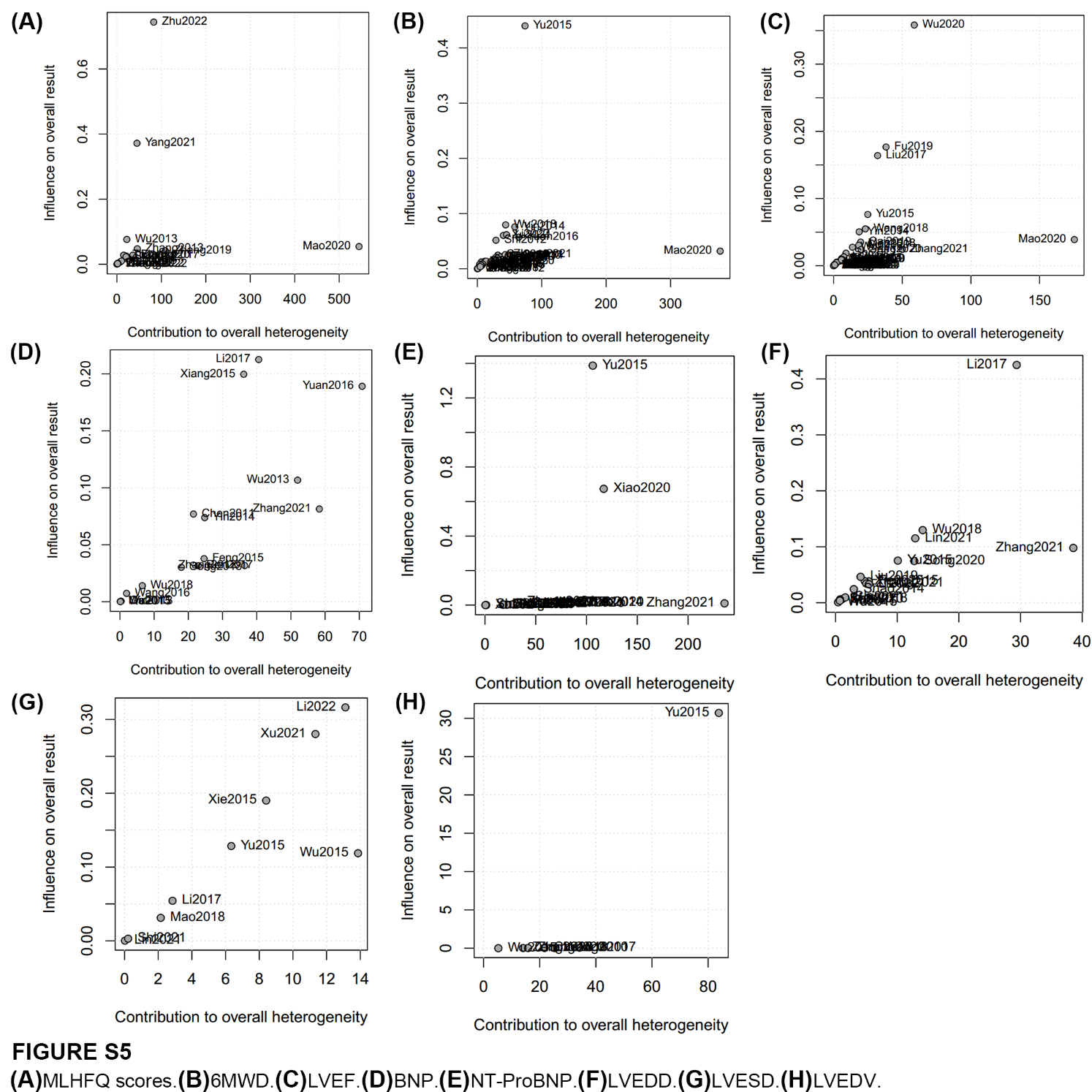

Supplement: Supplementary file 1 [file Table1.docx]
